# Safety of Ancestral Monovalent BNT162b2, mRNA-1273, and NVX-CoV2373 COVID-19 Vaccines in US Children Aged 6 Months to 17 Years

**DOI:** 10.1001/jamanetworkopen.2024.8192

**Published:** 2024-04-24

**Authors:** Mao Hu, Azadeh Shoaibi, Yuhui Feng, Patricia C. Lloyd, Hui Lee Wong, Elizabeth R. Smith, Kandace L. Amend, Annemarie Kline, Daniel C. Beachler, Joann F. Gruber, Mahasweta Mitra, John D. Seeger, Charlalynn Harris, Alex Secora, Joyce Obidi, Jing Wang, Jennifer Song, Cheryl N. McMahill-Walraven, Christian Reich, Rowan McEvoy, Rose Do, Yoganand Chillarige, Robin Clifford, Danielle D. Cooper, Richard A. Forshee, Steven A. Anderson

**Affiliations:** 1 Acumen LLC, Burlingame, California; 2 US Food and Drug Administration, Silver Spring, Maryland; 3 Optum Epidemiology, Boston, Massachusetts; 4 CVS Health/Aetna, Blue Bell, Pennsylvania; 5 Carelon Research, Wilmington, Delaware; 6 IQVIA, Falls Church, Virginia

## Abstract

**Importance:**

Active monitoring of health outcomes after COVID-19 vaccination provides early detection of rare outcomes that may not be identified in prelicensure trials.

**Objective:**

To conduct near-real-time monitoring of health outcomes after COVID-19 vaccination in the US pediatric population.

**Design, Setting, and Participants:**

This cohort study evaluated 21 prespecified health outcomes after exposure before early 2023 to BNT162b2, mRNA-1273, or NVX-CoV2373 ancestral monovalent COVID-19 vaccines in children aged 6 months to 17 years by applying a near-real-time monitoring framework using health care data from 3 commercial claims databases in the US (Optum [through April 2023], Carelon Research [through March 2023], and CVS Health [through February 2023]). Increased rates of each outcome after vaccination were compared with annual historical rates from January 1 to December 31, 2019, and January 1 to December 31, 2020, as well as between April 1 and December 31, 2020.

**Exposure:**

Receipt of an ancestral monovalent BNT162b2, mRNA-1273, or NVX-CoV2373 COVID-19 vaccine dose identified through administrative claims data linked with Immunization Information Systems data.

**Main Outcomes and Measures:**

Twenty-one prespecified health outcomes, of which 15 underwent sequential testing and 6 were only monitored descriptively due to lack of historical rates.

**Results:**

Among 4 102 016 vaccinated enrollees aged 6 months to 17 years, 2 058 142 (50.2%) were male and 3 901 370 (95.1%) lived in an urban area. Thirteen of 15 sequentially tested outcomes did not meet the threshold for a statistical signal. Statistical signals were detected for myocarditis or pericarditis after BNT162b2 vaccination in children aged 12 to 17 years and seizure after vaccination with BNT162b2 and mRNA-1273 in children aged 2 to 4 or 5 years. However, in post hoc sensitivity analyses, a statistical signal for seizure was observed only after mRNA-1273 when 2019 background rates were selected; no statistical signal was observed when 2022 rates were selected.

**Conclusions and Relevance:**

In this cohort study of pediatric enrollees across 3 commercial health insurance databases, statistical signals detected for myocarditis or pericarditis after BNT162b2 (ages 12-17 years) were consistent with previous reports, and seizures after BNT162b2 (ages 2-4 years) and mRNA-1273 vaccinations (ages 2-5 years) should be further investigated in a robust epidemiologic study with confounding adjustment. The US Food and Drug Administration concludes that the known and potential benefits of COVID-19 vaccination outweigh the known and potential risks of COVID-19 infection.

## Key Points

**Question:**

Were statistical signals detected for health outcomes after ancestral monovalent COVID-19 vaccination in US children aged 6 months to 17 years?

**Findings:**

In this cohort study of 4 102 106 vaccinated enrollees, 2 outcomes met the statistical threshold for a signal compared with historical rates: myocarditis or pericarditis after BNT162b2 (ages 12-17 years) and seizure after BNT162b2 (ages 2-4 years) and mRNA-1273 (ages 2-5 years) vaccinations.

**Meaning:**

Near-real-time monitoring detected a previously identified statistical signal for myocarditis or pericarditis and a new statistical signal for seizure; however, these results should be interpreted cautiously because the methods only screened for potential statistical signals and have several limitations.

## Teaser

This cohort study examines prespecified health outcomes after vaccination with an ancestral monovalent COVID-19 vaccine in US children aged 6 months to 17 years.

## Introduction

Three vaccines against COVID-19 are currently available for use in children in the US, including the BNT162b2 (Pfizer-BioNTech) and mRNA-1273 (Moderna) COVID-19 messenger RNA (mRNA) vaccines for those aged 6 months to 17 years and the protein-based NVX-CoV2373 (Novavax) vaccine for those aged 12 to 17 years.^1,2,3^ As of May 2023, the Centers for Disease Control and Prevention (CDC) reported that 31.78 million children had received at least 1 COVID-19 vaccine dose and 26.2 million children had completed the primary series of approximately 73 million children aged 6 months to 17 years in the US.^4^ The US Food and Drug Administration (FDA), utilizing the Biologics Effectiveness and Safety (BEST) Initiative, has been monitoring the safety of COVID-19 vaccines in children since their authorization by applying a near-real-time monitoring framework to evaluate the safety of COVID-19 vaccines. The near-real-time surveillance process is a signal detection or screening method and the first step in safety monitoring of these vaccines. This framework is designed to be sensitive enough to rapidly detect less common safety concerns as data accrue. However, results of such a study design do not establish a causal relationship between the vaccines and health outcomes and need to be interpreted with caution because of the limited adjustment for confounding and other forms of bias.

The BEST analysis of COVID-19 vaccines in children initially focused on the BNT162b2 COVID-19 ancestral monovalent vaccine authorized for use in children aged 5 to 17 years. Results of the initial, more limited, safety surveillance in children have been previously published.^5^ Surveillance has since been expanded as additional pediatric age groups and vaccine brands received authorization through late 2022. We present results from the expanded monitoring of health outcomes in children after exposure to the ancestral monovalent COVID-19 vaccines in the US targeting the original COVID-19 strain. This report presents final data from near-real-time monitoring, including additional age groups (6 months to 5 years of age), brands (mRNA-1273 and NVX-CoV2373), and more complete data accrual relative to the interim report.

## Methods

### Design

This study evaluated 21 prespecified health outcomes after exposure to BNT162b2, mRNA-1273, or NVX-CoV2373 ancestral monovalent COVID-19 vaccines in children aged 6 months to 17 years by applying a near-real-time monitoring framework using health care data from 3 commercial claims databases in the US. Fifteen outcomes underwent sequential testing, and 6 outcomes were only monitored descriptively due to lack of historical rates. This surveillance activity was conducted as part of the FDA public health surveillance mandate. Our study followed the Strengthening the Reporting of Observational Studies in Epidemiology (STROBE) reporting guideline for cohort studies. The study was conducted as part of a US FDA public health surveillance mandate under the BEST Initiative, which is part of the sentinel initiative. These safety surveillance activities are not considered research and therefore are exempt from institutional review board review and approval according to the requirements of 45 CFR §46;46.104(d)(2) under the 2018 Common Rule.^6^

### Data Sources

The study used commercial administrative health claims data from Optum (UnitedHealth and affiliated health plans), Carelon Research (Elevance Health, formerly Anthem, and affiliated health plans), and CVS Health (Aetna and affiliated health plans) containing longitudinal medical and pharmacy claims data supplemented with vaccination data from participating local and state Immunization Information Systems (eTable 1 in Supplement 1).^7^ These data sources are nationally representative of the commercially insured population aged 0 to 64 years and provide comprehensive capture of medical services submitted for insurance reimbursement.

### Study Population and Period

The study included pediatric enrollees aged 6 months to 17 years who received an ancestral monovalent COVID-19 vaccine from the earliest date of its Emergency Use Authorization by age group through April 2023 (Optum), March 2023 (Carelon Research), and February 2023 (CVS Health) (eTable 2 in Supplement 1). The surveillance concluded on these respective dates because of limited accrual of exposures and health outcomes. The sequential analyses for most outcomes did not reach the expected number of events that was prespecified based on anticipated vaccination uptake in a 6-month period after authorization of individual vaccine products. Another contributing factor to halting surveillance was that the ancestral monovalent mRNA-1273 and BNT162b2 doses are no longer authorized for persons of all ages as of April 18, 2023.^8^

The inclusion criteria for the study included enrollment on the vaccination date and continuous enrollment in a participating medical health insurance plan from the start of an outcome-specific clean window (ie, the interval before vaccination during which a patient must not have had the outcome) to the COVID-19 vaccination date so that only new incident diagnoses of an outcome in the postvaccination risk window would contribute to the analysis (eTable 3 in Supplement 1).

### Exposures and Follow-Up

Exposure was defined as the receipt of a BNT162b2, mRNA-1273, or NVX-CoV2373 ancestral monovalent COVID-19 vaccine dose identified using brand and dose-specific *Current Procedural Terminology*/Healthcare Common Procedure Coding System codes,^9^ National Drug Codes, and CVX codes (eTable 4 in Supplement 1). Dose number was assigned based on the chronological order in which vaccinations were observed because administration codes were not available in pharmacy claims. The primary analyses included all follow-up time accrued after dose 1 and dose 2 combined. However, secondary analyses included stratification by dose number and follow-up time accrued after the individual dose (including monovalent third or booster doses) through censoring at subsequent vaccination, death, disenrollment, end-of-risk window, or end-of-study period.

### Health Outcomes

Twenty-one prespecified health outcomes were defined using claims-based algorithms (eTable 3 in Supplement 1). Outcomes were selected through clinical consultation and literature review.^10,11^ Considerations in the selection of the outcomes included serious events that have followed other immunizations or events that are potentially related to novel platforms or adjuvants. Fifteen health outcomes were assessed using sequential testing comparing rates with historical outcome rates (additional information on historical rates in the Sequential Testing section), and 6 were only monitored descriptively due to lack of sufficient sample size for historical rates.^11^ The outcome myocarditis, pericarditis, or co-occurring myocarditis and pericarditis (hereafter referred to as the outcome “myocarditis or pericarditis”) was assessed using 4 different definitions with varying risk windows and care settings (eTable 3 in Supplement 1) based on evidence from prior surveillance efforts and clinical input. The seizure outcome included generalized seizure and convulsions diagnosis codes (*International Statistical Classification of Diseases, Tenth Revision, Clinical Modification* [*ICD-10-CM*] codes R56.00, R56.01, and R56.9) and is hereafter referred to as seizure. We also descriptively monitored febrile seizures. This outcome was defined using the presence of *ICD-10-CM* code R56.00 or R56.01 and was a subset of those with a seizure outcome.

### Statistical Analysis

We estimated outcome rates in the vaccinated population stratified by age, sex, region, urban vs rural status, data source, and vaccine brand on a monthly basis. Race and ethnicity data are frequently missing within the databases and therefore were excluded from the analysis.

Monthly sequential testing was conducted using the Poisson Maximized Sequential Probability Ratio Test to detect statistical signals by generating the incidence rate ratio comparing outcome rates after vaccination with database-specific historical (expected) rates for 15 health outcomes.^12^ The comparison of rates after vaccination with historical (expected) outcome rates has limited control for confounding factors and is meant to be a rapid screening method. This method relies on repeated looks at data to determine whether there is a statistical increase due to observed rates of outcomes after vaccination that exceeds expected outcome rates. This is also known as a statistical signal.

We estimated annual historical rates from January 1 to December 31, 2019, and January 1 to December 31, 2020, as well as between April 1 and December 31, 2020. These rates had the advantage of being most proximal in time to our study period while representing the rates in a COVID-19 vaccine–naive population. Historical rates were adjusted for claims processing delay to account for observation delay and standardized by age and sex where case counts permitted.^13^ Selection of the historical comparator rate was based on the overlap between the 95% CIs for the periods before and during the COVID-19 pandemic. If rates in these historical periods differed substantially, we selected the minimum or more stable rate as a more conservative approach.

Tests were stratified by age based on age group–specific authorizations by vaccine brand as well as availability of background rates using historical comparator data. For BNT162b2, the age groups were 6 months to 4 years, 5 to 11 years, 12 to 15 years, and 16 to 17 years. For mRNA-1273, the age groups were 6 months to 5 years, 6 to 11 years, 12 to 15 years, and 16 to 17 years. For NVX-CoV2373, the age groups were 12 to 15 years and 16 to 17 years. For the seizure outcome, we further stratified the youngest age groups into 6 months to 1 year and 2 to 4 years for BNT162b2 and 6 months to 1 year and 2 to 5 years for mRNA-1273.

Sequential testing for each outcome commenced when a minimum of 3 cases accrued in the risk window. One-tailed tests were used with the null hypothesis that the observed rate was no greater than the historical comparator rate beyond a prespecified test margin for each outcome-dose-age group being sequentially tested, with an α level of 1%. A stringent α level was selected to increase the specificity of statistical signals detected from testing multiple outcomes across different analyses. The log likelihood ratio was calculated comparing the likelihood of the observed incidence rate ratio and the null hypothesis. At each test, if the log likelihood ratio exceeded a prespecified critical value, the null hypothesis was rejected, and a statistical signal was declared. Surveillance continued until a statistical signal was detected, the prespecified maximum surveillance length was reached, or the end of study period was reached.^11^

We conducted testing in each database separately but present aggregated results in this report for interpretability and due to privacy concerns with small counts in individual databases. We consider an outcome to have signaled if a statistical signal was seen in 1 or more databases.

Signal characterization was conducted after a statistical signal was identified to provide data quality assessment.^14^ We conducted quality checks to rule out database errors or changes in the patterns of diagnosis codes used to identify events in the study period and in the historical period, estimated the relative risk of outcomes within demographic strata by age and sex, examined the timing of outcomes occurrence during the prespecified risk window, and assessed whether the statistical signal was sensitive to changes in background rates selection by conducting a post hoc sensitivity analysis using 2019 and 2022 background rates as the historical comparator in sequential testing. Medical record review^15^ was conducted for the myocarditis or pericarditis outcome after identification of a statistical signal; methods and results are detailed in the eAppendix in Supplement 1.

## Results

### Descriptive Monitoring

The study included 4 102 016 vaccinated individuals aged 6 months to 17 years, with 3 920 563 (95.6%) receiving BNT162b2 vaccination, 174 427 (4.3%) receiving mRNA-1273, and 53 (<0.1%) receiving NVX-CoV2373 (Table). Demographic characteristics of the mRNA-vaccinated population except age reported at first dose were largely similar across vaccine brands. While a majority (93.4%) of BNT162b2-vaccinated individuals were 5 years or older, a majority (97.6%) of mRNA-1273–vaccinated individuals were younger than 5 years. Across all vaccine brands, 2 058 142 (50.2%) were male and 2 043 874 (49.8%) were female, and 3 901 370 (95.1%) lived in an urban area (Table). A majority of mRNA-1273 recipients were vaccinated in the office setting (71.3%), whereas the most frequent setting for BNT162b2 vaccination was the pharmacy setting (41.1%). A total of 8 444 355 ancestral monovalent COVID-19 vaccine doses were administered (Figure), including 8 121 591 BNT162b2 doses (dose 1: 3 843 778; dose 2: 3 235 442, dose 3 or monovalent booster: 1 033 036, and unknown or unclear: 9335), 322 628 mRNA-1273 doses (dose 1: 173 857; dose 2: 140 734; dose 3 or monovalent booster: 5284; and unknown or unclear: 2753) administered to children aged 6 months to 17 years, as well as 136 NVX-CoV2373 doses (dose 1: 63; dose 2: 43; dose 3 or monovalent booster and unknown or unclear: 30) administered to children aged 12 to 17 years (eTable 5 in Supplement 1). We observed low case counts for outcomes that were monitored descriptively only (<80 cases of any given outcome in all 3 databases combined) (eTable 6 in Supplement 1).

**Table.** Characteristics of Pediatric Population Receiving Ancestral Monovalent BNT162b2, mRNA-1273, and NVX-CoV2373 COVID-19 Vaccines From All Data Sources^a^

Characteristic^b^	No. (%) of persons vaccinated			
	BNT162b2 (n = 3 920 563)	mRNA-1273 (n = 176 427)	NVX-CoV2373 (n = 53)	Multiple brands (n = 4973)
Age at first dose				
6 mo-4 or 5 y^c^	256 958 (6.6)	172 161 (97.6)	0	2299 (46.2)
5 or 6-11 y^c^	1 558 895 (39.8)	1687 (1.0)	0	1471 (29.6)
12-15 y	1 389 351 (35.4)	1420 (0.8)	27 (50.9)	945 (19.0)
16-17 y	715 359 (18.2)	1159 (0.7)	26 (49.1)	258 (5.2)
Sex				
Female	1 954 260 (49.8)	86 588 (49.1)	24 (45.3)	2521 (50.7)
Male	1 965 853 (50.1)	89 808 (50.9)	29 (54.7)	2452 (49.3)
Missing or unknown	450 (0.01)	31 (0.02)	0	0
Urban vs rural				
Rural	191 550 (4.9)	4465 (2.5)	NR	NR
Urban	3 724 798 (95.0)	171 771 (97.4)	50 (94.3)	4751 (95.5)
Missing or unknown	4215 (0.1)	191 (0.1)	NR	NR
US Department of Health and Human Services region				
1 (Connecticut, Maine, New Hamphire, Rhode Island, and Vermont)	252 282 (6.4)	15 436 (8.8)	NR	287 (5.8)
2 (New Jersey, New York, Puerto Rico, and Virgin Islands)	481 296 (12.3)	25 927 (14.7)	NR	1844 (37.1)
3 (Delaware, Washington DC, Maryland, Pennsylvania, Virginia, and West Virginia)	421 390 (10.8)	21 765 (12.3)	NR	455 (9.2)
4 (Alabama, Florida, Georgia, Kentucky, Mississippi, North Carolina, South Carolina, and Tennessee)	610 942 (15.6)	16 869 (9.6)	NR	523 (10.5)
5 (Illinois, Indiana, Michigan, Minnesota, Ohio, and Wisconsin)	644 718 (16.4)	24 061 (13.6)	NR	465 (9.4)
6 (Arkansas, Louisianna, New Mexico, Oklahoma, and Texas)	330 374 (8.4)	11 142 (6.3)	NR	263 (5.3)
7 (Iowa, Kansas, Missouri, and Nebraska)	159 328 (4.1)	6401 (3.6)	NR	NR
8 (Colorado, Montana, North Dakota, South Dakota, Utah, and Wyoming)	168 549 (4.3)	6998 (4.0)	NR	203 (4.1)
9 (Arizona, California, Hawaii, Nevada, American Samoa, Commonwealth of the Northern Mariana Islands, Federated States of Micronesia, Guam, Marshall Islands, and Republic of Palau)	720 576 (18.4)	37 394 (21.2)	NR	621 (12.5)
10 (Alaska, Idaho, Oregon, and Washington)	127 558 (3.2)	10 260 (5.8)	NR	203 (4.1)
Missing or unknown	3550 (0.1)	174 (0.1)	0	NR
Facility type				
Hospital	194 886 (5.0)	11 492 (6.5)	NR	NR
Office	1 115 926 (28.5)	125 781 (71.3)	NR	3559 (71.6)
Pharmacy	1 610 036 (41.1)	9940 (5.6)	32 (60.4)	337 (6.8)
Skilled nursing facility	NR	0	0	0
Home health agency	NR	73 (0.04)	0	NR
Mass immunization center	110 794 (2.8)	2448 (1.4)	NR	121 (2.4)
Other	135 842 (3.5)	3851 (2.2)	NR	151 (3.0)
Missing or unknown	751 900 (19.2)	22 842 (13.0)	NR	710 (14.3)

Abbreviation: NR, not reported (data for sample sizes of 1 to 10 and that can be used to back-calculate small cell sizes were masked for confidentiality).

^a^ Data are combined from Optum through April 2023, Carelon Research through March 2023, and CVS Health through February 2023. Percentages may not total 100% due to rounding.

^b^ Patient characteristics were assessed at the time of first vaccine dose, which was dose 1 or dose 2 depending on the dose that met Emergency Use Authorization–specific dose eligibility criteria; persons who received a different brand of vaccine from their dose 1 are categorized in the multiple brands category.

^c^ Children aged 6 months to 4 years who were vaccinated with the BNT162b2 COVID-19 vaccine are included in 6 months to 4 or 5 years age group. Children aged 6 months to 5 years who were vaccinated with the mRNA-1273 COVID-19 vaccine are included in the 6 months to 4 or 5 years age group. Children aged 5 to 11 years who were vaccinated with the BNT162b2 COVID-19 vaccine are included in the 5 or 6 to 11 years age group. Children aged 6 to 11 years who were vaccinated with the mRNA-1273 COVID-19 vaccine are included in the 5 or 6 to 11 years age group.

**Figure.**
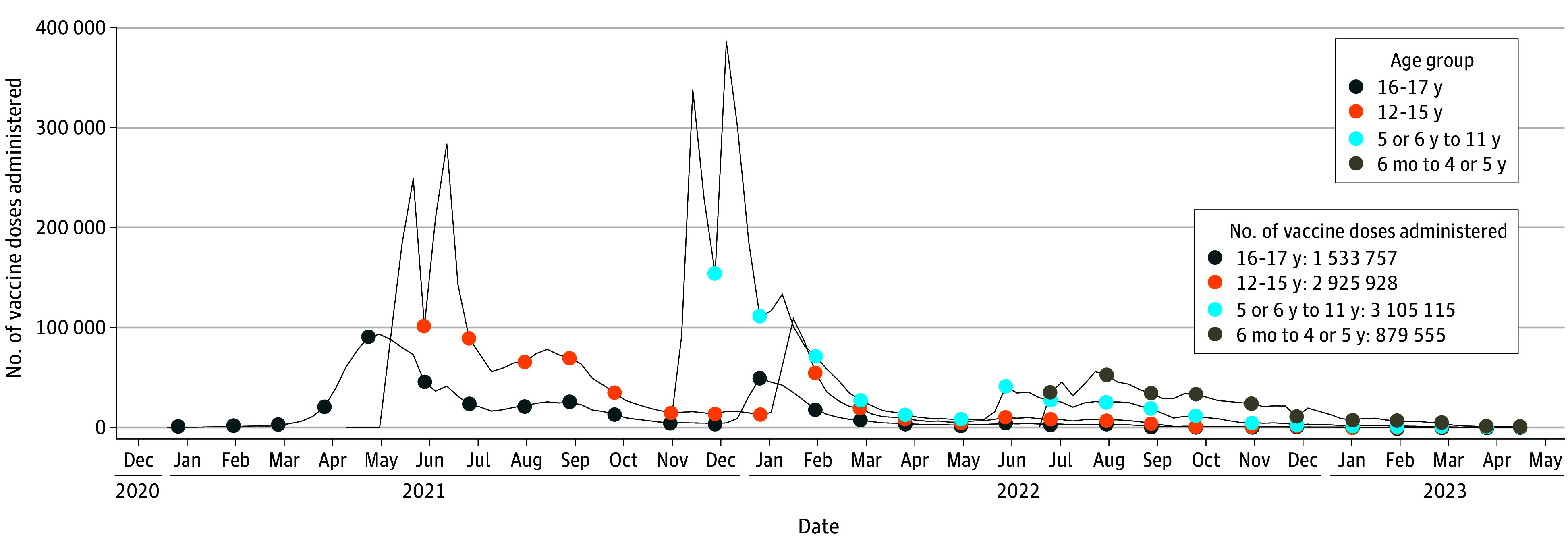
Distribution of Ancestral Monovalent Doses of BNT162b2, mRNA-1273, and NVX-CoV2373 COVID-19 Vaccines Administered Over Time by Age Group From All Data Sources Data are combined from Optum through April 2023, Carelon Research through March 2023, and CVS Health through February 2023.

### Sequential Testing

Among 15 outcomes that were sequentially tested, 2 outcomes met the statistical threshold for a signal, including myocarditis or pericarditis in children aged 12 to 15 years and 16 to 17 years and seizure in children aged 2 to 4 or 5 years. Statistical signals for myocarditis or pericarditis were identified in the primary analysis after BNT162b2 COVID-19 vaccination among children aged 12 to 15 years and 16 to 17 years in all 3 databases. Additionally, dose-specific statistical signals for 1 or more definitions of the outcomes were detected in children aged 12 to 17 years after dose 1, dose 2, and dose 3 of BNT162b2 vaccine in at least 1 of the 3 databases (eTable 8 in Supplement 1).

In the primary analysis, seizure met the statistical threshold for a signal in children aged 2 to 4 years after BNT162b2 vaccination in all 3 databases and in children aged 2 to 5 years after mRNA-1273 vaccination in 2 of the 3 databases. Dose-specific statistical signals for seizure were detected in 2 of the 3 databases after dose 1 and dose 2 BNT162b2 vaccination in children aged 2 to 4 years and after dose 2 of mRNA-1273 vaccination in children aged 2 to 5 years (eTable 8 in Supplement 1). Sequential testing was not initiated for any of the 15 outcomes for mRNA-1273 in children aged 5 to 17 years and NVX-CoV2373 in children aged 12 to 17 years because no outcomes were observed in any databases.

### Signal Characterization

Because the myocarditis or pericarditis outcome is a known adverse outcome after COVID-19 mRNA vaccinations, further signal characterization activities were not conducted. In the evaluation of the statistical signal for seizure among children aged 2 to 4 or 5 years, none of the prespecified data quality checks, including claims duplication, timing of events within prespecified risk window, and incidence rate ratio estimates, raised any data quality concerns.

There were 72 observed seizure cases among children aged 2 to 4 or 5 years, and 51 (70.8%) of these cases met the definition of febrile seizures (a subset of the seizure definition). No differences in rates of seizure by sex were identified. The timing of cases did not indicate substantial clustering with cases distributed across the 0- to 7-day risk window; 23 (31.9%) of the seizure cases occurred within the 0- to 1-day period after COVID-19 vaccination. The median (IQR) time between vaccination and diagnosis of seizure was 2 (1-5) days (eFigure in Supplement 1).

The statistical signal for seizure was sensitive to changes in the selection of comparator rates. Evaluation of the annual background rate of seizure indicated that the rates used in the primary analyses (2020) were lower than rates in 2022 and 2019. Background rates in 2022 and 2019 ranged from approximately 2.2 to 2.4 times and 1.7 to 1.9 times the 2020 rates, respectively, across 3 databases (eTable 7 in Supplement 1). In the post hoc sensitivity analysis, using 2022 background rates as the comparator in sequential testing did not identify any statistical signals for seizure in any databases. Using 2019 background rates as the comparator resulted in a statistical signal for seizure after primary series vaccination with mRNA-1273 in 2 of the 3 databases and after dose 2 vaccination with mRNA-1273 in 1 of the 3 databases.

## Discussion

Our near-real-time monitoring of 21 prespecified health outcomes after ancestral monovalent COVID-19 vaccines detected statistical signals for myocarditis or pericarditis in the 12- to 17-year age group and seizure in the 2- to 4- or 5-year age group. We did not detect statistical signals for other outcomes that were sequentially tested.

The myocarditis or pericarditis statistical signal is consistent with peer-reviewed reports^16,17,18^ demonstrating an elevated risk of this outcome after mRNA vaccines among younger males aged 12 to 29 years. Myocarditis and pericarditis are rare events, with a reported mean incidence of 39.3 cases per 1 million vaccine doses administered in children aged 5 to 17 years within 7 days after BNT162b2 vaccination.^19,20^ For the myocarditis or pericarditis outcome measured in the inpatient and emergency department settings in the 1 to 7 days after vaccinations, we observed approximately 27.0 inpatient or emergency department cases per million doses in days 1 to 7 after the primary series in children aged 12 to 15 years and 38.2 cases per million doses after the primary series in children aged 16 to 17 years. We did not detect a statistical signal for myocarditis or pericarditis in children younger than 12 years, which is consistent with reports from other surveillance systems.^21,22^

The statistical signal for seizure in children aged 2 to 4 or 5 years has not been previously reported for this age group in active surveillance studies of mRNA COVID-19 vaccines. However, there are reports from the Vaccine Adverse Events Reporting System (VAERS) database, which is a passive reporting system and has limitations. In an analysis of VAERS data, only 8 seizures were identified after approximately 1 million mRNA vaccinations through August 2022 in children aged 6 months to 5 years. Six of the 8 seizures were afebrile on medical evaluation.^23^ Prelicensure vaccine safety data indicate that among young children, seizure after mRNA COVID-19 vaccines are rare; a clinical trial of 3013 BNT162b2 vaccine recipients in children aged 6 months to 4 years reported only 5 febrile convulsions cases, and only 1 of those (in a 6-month-old participant) was considered possibly related to the vaccine or may also have been caused by a concurrent viral infection.^24^ Among studies conducted in children with childhood epilepsy (age <18 years), no increased risk of medically attended seizures was identified after immunization with COVID-19 vaccines. Seizure risk after COVID-19 vaccination was lower in children who were seizure free for more than 6 months before vaccination. However, the incidence of general adverse events after vaccination was low, with no severe adverse events recorded.^25,26^ Generally, there is limited evidence linking the mRNA COVID-19 vaccines to a seizure onset among vaccinated children aged 2 to 4 or 5 years.

The new statistical signal for seizure observed in our study should be interpreted with caution and further investigated in a more robust epidemiologic study. Our study used a broad seizure outcome definition with a 0- to 7-day risk window because of its applicability to older children. However, in children younger than 5 years, vaccine-related seizures typically manifest as a febrile seizure.^27^ Although most seizure cases met the febrile seizure definition, there was no statistically significant clustering observed at days 0 to 1. Because febrile seizures can be common in young children for a variety of reasons, the analysis may have identified febrile seizures unrelated to the vaccination later in the risk window.

Our post hoc sensitivity analysis suggests that our results are sensitive to comparator rate selection. We decided to use 2020 seizure rates as comparators to maximize sensitivity in the primary analysis. However, seizure rates were substantially lower in 2020 than in 2019 or 2022. There may be a few reasons for the observed differences in background rates. Pandemic-related interventions may have resulted in a lower seizure rate in 2020. The CDC reports that weekly seizure- or epilepsy-related emergency department visits decreased sharply during the early pandemic period (2020) among all age groups, especially children aged 0 to 9 years, which could explain the higher rates in 2019 and 2022 compared with 2020 during the early pandemic when health care resource limitations and other pandemic-related interventions may have impacted rates of pediatric illnesses.^28^ Our analysis suggests that using 2019 rates as the comparator would result in a statistical signal for mRNA-1273 only. However, there is evidence that 2019 may also be a sensitive choice for comparator year due to elevated rates in 2022. Seizure rates may be elevated in 2022 compared with 2019 and 2020 because of an increased incidence of respiratory infections (influenza and respiratory syncytial virus), which are shown to be associated with febrile seizure in younger children, during the study period (mid-2022 to mid-2023) compared with earlier years.^29,30,31,32^ Our analysis suggests that no statistical signals would be observed when using 2022 rates as the comparator.

### Strengths and Limitations

Our study has a number of strengths. The study included a large, geographically diverse population from 3 US commercial health insurance databases. Due to availability of more complete information from claims supplemented with Immunization Information Systems data and a short data lag from health encounters, we monitored ancestral monovalent COVID-19 vaccines safety in a near-real-time manner. Additionally, a subset of the identified myocarditis or pericarditis cases were confirmed through medical record review.

The study also has some limitations. First, we used a near-real-time surveillance method, which may be sensitive to comparator rate selection and does not include controlling for bias and confounding. Therefore, results from this study do not establish a causal relationship between the vaccines and health outcomes, and statistical signals should be further evaluated. Second, this study only includes data from a commercially insured pediatric population and may not be nationally representative. Third, small counts of NVX-CoV2373 prevented evaluation of most demographic factors due to privacy concerns. Fourth, we could not conduct medical record review for all outcomes included in the study due to resource, time, and legal constraints. For the myocarditis or pericarditis outcome, we reviewed medical records of a subset of identified cases of myocarditis or pericarditis that were obtained from the medical professionals. Medical record review of the seizure outcome is under way. Fifth, there are limitations as a result of selecting 2020 background rates as the historical comparator for the seizure analysis because this period was marked by behavioral shifts during the early pandemic that may have caused a sustained decrease in the underlying outcome rates. For that reason, we compared both prepandemic and peripandemic periods across data partners when selecting historical rates and generally selected the lower rate as the historical comparator. This approach was taken to increase sensitivity of our tests and enable rapid monitoring; however, further evaluation of the statistical signal for seizure will use a self-controlled design to reduce confounding bias.^33^ Although each dose was analyzed separately, we grouped only doses 1 and 2 together to prioritize rapid signal detection, which may have limited our ability to detect a signal for the BNT162b2 3-dose primary series for children aged 6 months to 4 years. Inclusion of dose 3 would have led to longer surveillance, slower α spending, and potential delays in detection of statistical signals. Sixth, there were only 53 children aged 12 to 17 years who received at least 1 dose of NVX-CoV2373 and 4266 children aged 5 to 17 years who received at least 1 dose of mRNA-1273. Consequently, it is not possible to draw any meaningful conclusions about these vaccines in these age groups based on this study.

## Conclusions

In this cohort study of pediatric enrollees in 3 commercial health insurance databases, we detected a statistical signal for myocarditis or pericarditis in older children, which is consistent with existing literature, and a new statistical signal for seizure in young children, which is being further evaluated in a more robust study. The FDA concludes that the known and potential benefits of COVID-19 vaccination outweigh the known and potential risks of COVID-19 infection. This study was conducted under the FDA BEST Initiative, which plays a major role in the larger US federal government vaccine safety monitoring efforts and further supports regulatory decision-making regarding COVID-19 vaccines.

## Notes

Supplement 1. **eTable 1.** Description of Database Characteristics **eTable 2.** Monovalent COVID-19 Vaccines Dosing Schedule Associated with Emergency Use Authorizations or Approval Description of Database Characteristics **eTable 3.** Outcomes, Age Groups, Settings, Clean Windows, Risk Windows, and Analysis Type for the BNT162b2, mRNA-1273, and NVX-CoV2373 COVID-19 Vaccinated Population (Ages 6 Months to 17 Years) **eTable 4.** Administrative Codes for COVID-19 Vaccine Administration Used in Claims and IIS Databases **eTable 5.** Vaccine Dose Counts for Monovalent BNT162b2, mRNA-1273, NVX-CoV2373 COVID-19 Vaccines Administered to the Pediatric Population Across All Data Sources **eTable 6.** Descriptive Summary of Outcomes Included in Descriptive Analysis Only **eTable 7.** Annual Rates of Seizures Per 100 000 Person-Years by Data Source for Children Aged 2-4 Years and 2-5 Years, 2020 (Baseline), 2019 and 2022 **eTable 8. **Sequential Testing Results for the Pediatric Population Receiving BNT162b2 or mRNA-1273 Ancestral Monovalent COVID-19 Vaccines Stratified by Vaccine Dose and Outcome **eFigure.** Distribution of Days Between Vaccination and Seizures (N = 72 Seizures Events) After Monovalent BNT162b2 (2-4 Years) and mRNA-1273 (Ages 2-5 Years) COVID-19 Vaccines **eAppendix.** Medical Record Review

Supplement 2.
Data Sharing Statement
