# Supplementary material for: Safety of Ancestral Monovalent BNT162b2, mRNA-1273, and NVX-CoV2373 COVID-19 Vaccines in US Children Aged 6 Months to 17 Years
Source: JAMA Netw Open. 2024 Apr 24;7(4):e248192. doi: 10.1001/jamanetworkopen.2024.8192 (PMC11043896; doi:10.1001/jamanetworkopen.2024.8192)
Supplement: Supplement 1. — eTable 1. Description of Database Characteristics eTable 2. Monovalent COVID-19 Vaccines Dosing Schedule Associated with Emergency Use Authorizations or Approval Description of Database Characteristics eTable 3. Outcomes, Age Groups, Settings, Clean Windows, Risk Windows, and Analysis Type for the BNT162b2, mRNA-1273, and NVX-CoV2373 COVID-19 Vaccinated Population (Ages 6 Months to 17 Years) eTable 4. Administrative Codes for COVID-19 Vaccine Administration Used in Claims and IIS Databases eTable 5. Vaccine Dose Counts for Monovalent BNT162b2, mRNA-1273, NVX-CoV2373 COVID-19 Vaccines Administered to the Pediatric Population Across All Data Sources eTable 6. Descriptive Summary of Outcomes Included in Descriptive Analysis Only eTable 7. Annual Rates of Seizures Per 100 000 Person-Years by Data Source for Children Aged 2-4 Years and 2-5 Years, 2020 (Baseline), 2019 and 2022 eTable 8. Sequential Testing Results for the Pediatric Population Receiving BNT162b2 or mRNA-1273 Ancestral Monovalent COVID-19 Vaccines Stratified by Vaccine Dose and Outcome eFigure. Distribution of Days Between Vaccination and Seizures (N = 72 Seizures Events) After Monovalent BNT162b2 (2-4 Years) and mRNA-1273 (Ages 2-5 Years) COVID-19 Vaccines eAppendix. Medical Record Review [file jamanetwopen-e248192-s001.pdf]

# Supplementary Online Content

Hu M, Shoaibi A, Feng Y, et al. Safety of ancestral monovalent BNT162b2, mRNA-1273, and NVX-CoV2373 COVID-19 vaccines in US children aged 6 months to 17 years. *JAMA Netw Open*. 2024;7(4):e248192. doi:10.1001/jamanetworkopen.2024.8192

**eTable 1**. Description of Database Characteristics
**eTable 2**. Monovalent COVID-19 Vaccines Dosing Schedule Associated with Emergency Use Authorizations or Approval
**eTable 3**. Outcomes, Age Groups, Settings, Clean Windows, Risk Windows, and Analysis Type for the BNT162b2, mRNA-1273, and NVX-CoV2373 COVID-19 Vaccinated Population (Ages 6 months to 17 years)
**eTable 4**. Administrative Codes for COVID-19 Vaccine Administration Used in Claims and IIS Databases
**eTable 5**. Vaccine Dose Counts for Monovalent BNT162b2, mRNA-1273, NVX-CoV2373 COVID-19 Vaccines Administered to the Pediatric Population Across All Data Sources
**eTable 6.** Descriptive Summary of Outcomes Included in Descriptive Analysis Only
**eTable 7.** Annual Rates of Seizures Per 100,000 Person-Years by Data Source for Children Aged 2-4 Years and 2-5 Years, 2020 (Baseline), 2019 and 2022
**eTable 8.** Sequential Testing Results for the Pediatric Population Receiving BNT162b2 or mRNA-1273 Ancestral Monovalent COVID-19 Vaccines Stratified by Vaccine Dose and Outcome
**eFigure.** Distribution of Days Between Vaccination and Seizures (N =72 Seizures Events) After Monovalent BNT162b2 (2-4 years) and mRNA-1273 (Ages 2-5 years) COVID-19 Vaccines
**eAppendix.** Medical Record Review

This supplementary material has been provided by the authors to give readers additional information about their work.

© 2024 Hu M et al. *JAMA Network Open.*

**eTable 1. Description of Database Characteristics**

| Database | Description | Claims Type | Update frequency | Data Lag, Time to 80% Completeness[a] | Average No. Enrollees aged 6 months-17 years (2020-2023) [b] | No. IIS Jurisdictions Incorporated into Analysis[c] |
|---|---|---|---|---|---|---|
| CVS Health | CVS Health transforms Aetna health plan enrollment, demographic, and medical and drug claims data, for individuals enrolled from January 2018 forward. The Aetna data includes commercial, including Affordable Care act (ACA) Marketplace, and Medicare Advantage health plans into a patient-centered, comprehensive Common Data Model (CDM). | Fully Adjudicated | Monthly | ~ 3-4 months for IP claims, 2-3 months for OP claims, and 1-2 months for professional claims | 6 months-4 years: > 1.08m<br>5-11 years: > 1.47m<br>12-15 years: > 934k<br>16-17 years: > 514k | 23 |
| Optum Pre-adjudicated Claims | The Optum data includes enrollment, prescription drug and pre-adjudicated hospital and physician health insurance claims. The pre-adjudicated claims database includes claims for privately insured and Medicare Advantage enrollees. Hospital and physician claims undergo initial processing on a daily basis from a large number of providers across the US who accept patients with health insurance. | Pre-Adjudicated | Bi-Weekly | ~ 1-2 months for IP, OP, and professional claims | 6 months-4 years: > 839k<br>5-11 years: > 1.18m<br>12-15 years: > 750k<br>16-17 years: > 392k | 20 |
| Carelon Research | Carelon Research is a wholly-owned, research subsidiary of Elevance Health, Inc., a holding company owning several large US health plans associated with Anthem Blue Cross Blue Shield. Carelon has a US population database including individually insured by commercial and Medicare Advantage plans, the Healthcare Integrated Research Database (HIRD), with longitudinal data on health plan enrollees. | Fully Adjudicated | Monthly | ~ 2-3 months for IP claims and 1-2 months for OP and professional claims | 6 months-4 years: > 1.32m<br>5-11 years: > 1.86m<br>12-15 years: > 1.20m<br>16-17 years: > 647k | 9 |

[a] Data lag based on 2020 claims delay distribution

[b] Average number of annual enrollees in a given age category is reported for the years 2020-2023 except for Optum where the average is reported through 2021 based on the available data.

[c] IIS = Immunization Information System. IIS jurisdictions are used to supplement claims data in improving the capture of COVID-19 vaccine dose information. The FDA BEST Initiative, through its distributed data network, facilitated linkage of claims data with IIS data to enhance capture of vaccinations in insured populations. IIS jurisdictions were solicited to share COVID-19 vaccination data that were linked to individual-level claims records by individual commercial insurer data partners using personally identifiable information and IIS-specific linkage algorithms.

© 2024 Hu M et al. *JAMA Network Open.*

**eTable 2. Monovalent COVID-19 Vaccines Dosing Schedule Associated with Emergency Use Authorizations or Approval**

| Brand | Age Group (years) | Authorized dose | Date of Authorization/Approval |
|---|---|---|---|
| BNT162b2 | 6 months – 4 | Three-dose primary series (3 micrograms) | EUA: 06/17/2022[1] |
| | 5-11 | Two-dose primary series (10 micrograms) | EUA (primary series): 10/29/2021[2] |
| | | Third primary series dose for immunocompromised individuals (10 micrograms) | EUA (third dose): 01/03/2022[2] |
| | | Single booster at least 5 months after completion of primary series (10 micrograms) | EUA (single booster): 05/17/2022 [3] |
| | 12-15 | Two-dose primary series (30 micrograms) | EUA (primary series): 05/10/2021[4] |
| | | Third primary series dose for immunocompromised individuals (30 micrograms) | EUA (third dose): 08/12/2021[5] |
| | | First booster at least 5 months after completion of primary series (30 micrograms) | EUA (first booster): 01/03/2022[6] |
| | | Second booster at least 4 months after receipt of first booster for immunocompromised individuals (30 micrograms) | EUA (second booster): 03/29/2022[6] |
| | 16-17 | Two-dose primary series (30 micrograms) | Approval (primary series): 08/23/2021[7] EUA (primary series): 12/11/2020 [7] |
| | | Third primary series dose for immunocompromised individuals (30 micrograms) | EUA (third dose): 08/12/2021[5] |
| | | First booster at least 5 months after completion of primary series (30 micrograms) | EUA (first booster): 12/9/2021[6] |

© 2024 Hu M et al. *JAMA Network Open.*

| Brand | Age Group (years) | Authorized dose | Date of Authorization/Approval |
|---|---|---|---|
| | | Second booster at least 4 months after receipt of first booster for immunocompromised individuals (30 micrograms) | EUA (second booster): 03/29/2022[6] |
| mRNA-1273 | 6 months – 5 | Two-dose primary series (25 micrograms) | EUA (primary series): 06/17/2022[8] |
| | | Third primary series dose for immunocompromised individuals (25 micrograms) | EUA (third dose): 06/17/2022[8] |
| | 6-11 | Two-dose primary series (50 micrograms) | EUA (primary series): 06/17/2022[8] |
| | | Third primary series dose for immunocompromised individuals (50 micrograms) | EUA (third dose): 06/17/2022[8] |
| | 12-15 | Two-dose primary series (100 micrograms) | EUA (primary series): 06/17/2022[8] |
| | | Third primary series dose for immunocompromised individuals (100 micrograms) | EUA (third dose): 06/17/2022[8] |
| | 16-17 | Two-dose primary series (100 micrograms) | EUA (primary series): 06/17/2022[8] |
| | | Third primary series dose for immunocompromised individuals (100 micrograms) | EUA (third dose): 06/17/2022[8] |
| NVX-CoV2373 | 12-17 | Two dose primary series (5 micrograms) | EUA (primary series): 08/19/2022[9] |

© 2024 Hu M et al. *JAMA Network Open.*

**eTable 3. Outcomes, Settings, Clean Windows, Risk Windows, and Analysis Type for the BNT162b2, mRNA-1273, and NVX-CoV2373 COVID-19 Vaccinated Population (Ages 6 months to 17 years)**

| Outcomes[a] | Care Setting | Clean Window[b] | Risk Window | Analysis Type[d] |
|---|---|---|---|---|
| Acute Myocardial Infarction | IP | 365 days | 1-28 days [10,11] | Descriptive Only |
| Anaphylaxis | IP, OP-ED | 30 days | 0-1 day [12,13] | Descriptive and Sequential Testing |
| Appendicitis | IP, OP-ED | 365 days | 1-42 days [14,15] | Descriptive and Sequential Testing |
| Bell's Palsy | IP, OP, PB | 183 days | 1-42 days [16] | Descriptive and Sequential Testing |
| Common Site Thrombosis with Thrombocytopenia | [Defined in Footnote] [e] | 365 days | 1-28 days [17] | Descriptive and Sequential Testing |
| Deep Vein Thrombosis | IP, OP, PB | 365 days | 1-28 days [18,19] | Descriptive and Sequential Testing |
| Disseminated Intravascular Coagulation | IP, OP-ED | 365 days | 1-28 days [20] | Descriptive and Sequential Testing |
| Encephalitis or Encephalomyelitis | IP | 183 days | 1-42 days [21] | Descriptive and Sequential Testing |
| Febrile Seizures | IP, OP PB | 42 days | 0-1 days[c] | Descriptive Only |
| Guillain-Barré Syndrome | IP – Primary Position Only | 365 days | 1-42 days [22,23] | Descriptive and Sequential Testing[f] |
| Hemorrhagic Stroke | IP | 365 days | 1-28 days [10,11] | Descriptive and Sequential Testing[f] |
| Immune Thrombocytopenia | IP, OP, PB | 365 days | 1-42 days [24,25] | Descriptive and Sequential Testing |
| Kawasaki Disease | IP, OP, PB | 365 days | 1-28 days[c] | Descriptive Only |
| Multisystem Inflammatory Syndrome in Children | IP, OP-ED | 365 days | 1-42 days [26] | Descriptive Only |
| Myocarditis/Pericarditis (All Settings) (1-7 day) | IP, OP, PB | 365 days | 1-7 days [27] | Descriptive and Sequential Testing |
| Myocarditis/Pericarditis (All Settings) (1-21 day) | IP, OP, PB | 365 days | 1-21 days [28] | Descriptive and Sequential Testing |
| Myocarditis/Pericarditis (IP/OP-ED) (1-7 day) | IP, OP-ED | 365 days | 1-7 days [27] | Descriptive and Sequential Testing |
| Myocarditis/Pericarditis (IP/OP-ED) (1-21 day) | IP, OP-ED | 365 days | 1-21 days [28] | Descriptive and Sequential Testing |
| Narcolepsy | IP, OP, PB | 365 days | 1-42 days [29-31] | Descriptive and |

© 2024 Hu M et al. *JAMA Network Open.*

| Outcomes[a] | Care Setting | Clean Window[b] | Risk Window | Analysis Type[d] |
|---|---|---|---|---|
| | | | | Sequential Testing[f] |
| Non-Hemorrhagic Stroke | IP | 365 days | 1-28 days [10, 11] | Descriptive and Sequential Testing |
| Pulmonary Embolism | IP, OP, PB | 365 days | 1-28 days [18,19,32-35] | Descriptive and Sequential Testing |
| Seizures | IP, OP-ED | 42 days | 0-7 days [33] | Descriptive and Sequential Testing |
| Transverse Myelitis | IP, OP-ED | 365 days | 1-42 days [34] | Descriptive Only |
| Unusual Site Thrombosis with Thrombocytopenia | [Defined in Footnote] [e] | 365 days | 1-28 days [35] | Descriptive Only |

**Definitions**:

- **Clean Window** is the interval used to define incident outcomes where an individual enters the study cohort only if the health outcome of interest did not occur during that interval.
- **Risk Window** is the interval during which occurrence of the health outcome of interest will be included in the analyses.
- **Care Setting:** IP refers to inpatient facility claims. OP-ED refers to a subset of outpatient facility claims occurring in the emergency department. OP/PB refers to all outpatient facility claims, and professional/provider claims except those professional/provider claims with a laboratory place of service

[a] Source of the claims-based algorithm: FDA. Appendix 7. Safety AE Codes. https://www.bestinitiative.org/wp-content/uploads/2022/01/C19-Vax-Safety-AESI-Bkgd-Rate-Est-Protocol-Suppl.xlsx

[b] The clean window is the interval prior to vaccination where a patient must not have had the outcome.

[c] References for the duration of these windows could not be located in the literature and are instead based on input from clinicians.

[d] Sequential testing was conducted only when the minimum threshold of observed events was met (at least 3 events). For NVX-CoV2373 (ages 12 to 17 years) and mRNA-1273 (ages 6 to 17 years) sequential testing did not initiate due to not meeting this minimum threshold for any of the outcomes specified above

[e] Both Common thrombosis with thrombocytopenia and Unusual site thrombosis (broad) with thrombocytopenia are combined outcomes consisting of a thrombotic event (made up of other events such as acute myocardial infarction, deep vein thrombosis etc.,) and a thrombocytopenia event (defined in the IP, OP/PB setting). The overall setting definition for each outcome depends on individual setting definitions for each of these components.

[f] Feasibility of sequential testing of certain outcomes in specific age groups was evaluated based on availability of background rates. Guillain-Barré Syndrome and Hemorrhagic Stroke were sequentially tested in ages <5 years only and Narcolepsy in ages >5 years old only due to lack of estimable background rates in other age groups

© 2024 Hu M et al. *JAMA Network Open.*

**eTable 4. Administrative Codes for COVID-19 Vaccine Administration used in claims and IIS databases**

| HCPCS/CPT[a] Codes | CVX[b] Codes (IIS-Specific) | Manufacturer | Name | Age Group (years) | Vaccine Administration Code | NDC[c] 11 Labeler Product ID (Vial) | Dosing Interval |
|---|---|---|---|---|---|---|---|
| 91308 | 219 | Pfizer | Pfizer-BioNTech COVID-19 Vaccine | 6 m- 4 | 0081A (1st dose)<br>0082A (second dose)<br>0083A (third dose) | 59267-0078-01<br>59267-0078-04 | • 21+ days between dose 1 and dose 2<br>• 56+ days between dose 2 and dose 3 |
| 91307 | 218 | Pfizer | Pfizer-BioNTech COVID-19 Pediatric Vaccine | 5-11 | 0071A (1st dose)<br>0072A (2nd dose)<br>0073A (3rd dose)<br>0074A (booster dose) | 59267-1055-0159267-1055-02<br>59267-1055-04 | • 21+ days between dose 1 and dose 2<br>• For immunocompromised, 21+ days between dose 1 and dose 2, and 28+ days between dose 2 and additional primary dose (dose 3) |
| 91305 | 217 | Pfizer | Pfizer-BioNTech COVID-19 Vaccine | 12-17 | 0051A (1st dose)<br>0052A (2nd dose)<br>0053A (3rd dose)<br>0054A (booster dose) | 59267-1025-01<br>59267-1025-02<br>59267-1025-03<br>59267-1025-04 | • 21+ days between dose 1 and dose 2 and 5+ months between dose 2 and third/booster dose<br>• For immunocompromised, 21+ days between dose 1 and dose 2, 28+ days between dose 2 and dose 3. Additionally, booster dose recommended 3+ months after primary series |
| 91300 | 208 | Pfizer | Pfizer-BioNTech COVID-19 Vaccine | 12-17 | 0001A (1st dose)<br>0002A (2nd dose)<br>0003A (3rd dose) | 59267-1000-01<br>59267-1000-02<br>59267-1000-03 | • 21+ days between dose 1 and dose 2 and 5+ months between dose 2 and third/booster dose<br>• For |

© 2024 Hu M et al. *JAMA Network Open.*

| HCPCS/CPT[a] Codes | CVX[b] Codes (IIS-Specific) | Manufacturer | Name | Age Group (years) | Vaccine Administration Code | NDC[c] 11 Labeler Product ID (Vial) | Dosing Interval |
|---|---|---|---|---|---|---|---|
| | | | | | 0004A (booster dose)<br>0002A (2nd dose)<br>0003A (3rd dose)<br>0004A (booster dose) | | immunocompromised, 21+ days between dose 1 and dose 2, 28+ days between dose 2 and dose 3. Additionally, booster dose recommended 3+ months after primary series |
| 91301, 91306, 91309 | 207 | Moderna | Moderna COVID-19 Vaccine | 6 m-17 | 0011A, 0091A (1st dose)<br>0012A, 0092A (2nd dose)<br>0013A, 0093A (3rd dose)<br>0064A, 0094A (booster dose) | 61434-0043-00<br>61434-0043-01<br>80777-0100-11<br>80777-0100-98<br>80777-0100-99<br>80777-0277-05<br>80777-0279-05<br>80777-0273-98<br>80777-0273-10<br>80777-0273-99<br>80777-0273-15<br>80777-0273-98<br>80777-0273-05<br>80777-0273-99 | • 28+ days between dose 1 and dose 2<br>• For immunocompromised, 28 days between dose 1 and dose 2, and 28+ days between dose 2 and dose 3. |
| 91304 | 211 | Novavax | Novavax COVID-19 Vaccine | 12-17 | 0041A (1st dose)<br>0042A (2nd dose) | 80631-0100-01<br>80631-0100-10 | • 21+ days between dose 1 and dose 2 including for immunocompromised |

[a] HCPCS: The Healthcare Common Procedure Coding System produced by the Centers for Medicare and Medicaid Services (CMS)[36]
CPT: The Current Procedural Terminology codes[36]
[b] CVX: Vaccine administered code set developed and maintained by the Centers for Disease Control and Prevention's (CDC) National Center of Immunization and Respiratory Diseases (NCIRD)[37]
[c] NDC: National Drug Codes used to identify and report drugs using a unique, three-segment number which serves as the Food and Drug Administration's (FDA) identifier for drugs[38]

© 2024 Hu M et al. *JAMA Network Open.*

**eTable 5**. **Vaccine Dose Counts for Monovalent BNT162b2, mRNA-1273, NVX-CoV2373 COVID-19 Vaccines Administered to the Pediatric Population Across All Data Sources[a]**

| Vaccine Dose | BNT162b2 | | mRNA-1273 | | NVX-CoV2373 | |
|---|---|---|---|---|---|---|
| | N | % | N | % | N | % |
| **Total** | 8,121,591 | 100 | 322,628 | 100 | 136 | 100 |
| **Dose 1** | 3,843,778 | 47.33 | 173,857 | 53.89 | 63 | 46.32 |
| **Dose 2** | 3,235,442 | 39.84 | 140,734 | 43.62 | 43 | 31.62 |
| **Dose 3/Monovalent Booster** | 1,033,036 | 12.72 | 5,284 | 1.64 | § | § |
| **Unknown/Unclear** | 9,335 | 0.11 | 2,753 | 0.85 | § | § |

[a] Combines Optum data through 4/2023, Carelon Research data through 3/2023, CVS Health data through 2/2023
§ Cell sizes 1-10 and cells that can be used to back-calculate small cell sizes were masked for confidentiality

© 2024 Hu M et al. *JAMA Network Open.*

**eTable 6. Descriptive Summary of Outcomes Included in Descriptive Analysis Only**

| Outcome | Brand | All Data Sources | | Carelon Research[a] | | CVS Health[b] | | Optum[c] | |
|---|---|---|---|---|---|---|---|---|---|
| | | No. Vaccine Doses | No. Outcomes | No. Vaccine Doses | No. Outcomes | No. Vaccine Doses | No. Outcomes | No. Vaccine Doses | No. Outcomes |
| Acute myocardial infarction | BNT162b2 | 6,650,342 | § | 2,589,974 | § | 2,120,805 | § | 1,939,563 | 0 |
| | mRNA-1273 | 252,959 | 0 | 94,878 | 0 | 85,473 | 0 | 72,608 | 0 |
| | NVX-CoV2373 | 98 | 0 | 18 | 0 | 18 | 0 | 62 | 0 |
| Febrile Seizure | BNT162b2 | 7,932,684 | 76 | 3,064,741 | 26 | 2,565,776 | 27 | 2,302,167 | 23 |
| | mRNA-1273 | 315,520 | 37 | 119,174 | 14 | 108,685 | § | 87,661 | § |
| | NVX-CoV2373 | 133 | 0 | 21 | 0 | 28 | 0 | 84 | 0 |
| Guillain-Barré syndrome | BNT162b2 | 6,650,324 | § | 2,589,971 | § | 2,120,793 | § | 1,939,560 | § |
| | mRNA-1273 | 252,959 | 0 | 94,878 | 0 | 85,473 | 0 | 72,608 | 0 |
| | NVX-CoV2373 | 98 | 0 | 18 | 0 | 18 | 0 | 62 | 0 |
| Hemorrhagic stroke | BNT162b2 | 6,650,157 | 14 | 2,589,902 | § | 2,120,744 | § | 1,939,511 | § |
| | mRNA-1273 | 252,950 | 0 | 94,874 | 0 | 85,473 | 0 | 72,603 | 0 |
| | NVX-CoV2373 | 98 | 0 | 18 | 0 | 18 | 0 | 62 | 0 |
| Kawasaki disease | BNT162b2 | 6,648,712 | 79 | 2,589,367 | 26 | 2,120,267 | 23 | 1,939,078 | 30 |
| | mRNA-1273 | 252,810 | § | 94,809 | § | 85,441 | § | 72,560 | § |
| | NVX-CoV2373 | 98 | 0 | 18 | 0 | 18 | 0 | 62 | 0 |
| Multisystem inflammatory syndrome in children (MIS-C) | BNT162b2 | 6,650,003 | 15 | 2,589,829 | § | 2,120,710 | § | 1,939,464 | § |
| | mRNA-1273 | 252,949 | 0 | 94,878 | 0 | 85,469 | 0 | 72,602 | 0 |
| | NVX-CoV2373 | 98 | 0 | 18 | 0 | 18 | 0 | 62 | 0 |
| Narcolepsy | BNT162b2 | 6,649,400 | 65 | 2,589,563 | 26 | 2,120,525 | 22 | 1,939,312 | 17 |
| | mRNA-1273 | 252,954 | 0 | 94,877 | 0 | 85,472 | 0 | 72,605 | 0 |
| | NVX-CoV2373 | 98 | 0 | 18 | 0 | 18 | 0 | 62 | 0 |
| Transverse myelitis | BNT162b2 | 6,650,321 | § | 2,589,967 | § | 2,120,801 | 0 | 1,939,553 | § |
| | mRNA-1273 | 252,959 | § | 94,878 | § | 85,473 | 0 | 72,608 | 0 |
| | NVX-CoV2373 | 98 | 0 | 18 | 0 | 18 | 0 | 62 | 0 |
| Unusual site thrombosis with thrombocytopenia | BNT162b2 | 6,650,303 | § | 2,589,961 | § | 2,120,793 | § | 1,939,549 | 0 |
| | mRNA-1273 | 252,959 | 0 | 94,878 | 0 | 85,473 | 0 | 72,608 | 0 |
| | NVX-CoV2373 | 98 | 0 | 18 | 0 | 18 | 0 | 62 | 0 |

[a] Data through 3/2023 [b] Data through 2/2023 [c] Data through 4/2023
§ Cell sizes 1-10 and cells that can be used to back-calculate small cell sizes were masked for confidentiality

© 2024 Hu M et al. *JAMA Network Open.*

**eTable 7. Background Annual Rate of Seizures Per 100,000 Person-Years by Data Source in Children Aged 2-4 Years and 2-5 Years, 2020 (Baseline), 2019 and 2022**

| | Ages 2-4 years | | | | | Ages 2-5 years | | | | |
|---|---|---|---|---|---|---|---|---|---|---|
| Data Source | **2020 (baseline)** | **2019[a]** | **2022[b]** | **Difference 2019 vs 2020 (Ratio)** | **Difference 2022 vs 2020 (Ratio)** | **2020 (baseline)** | **2019[a]** | **2022[b]** | **Difference 2019 vs 2020 (Ratio)** | **Difference 2022 vs 2020 (Ratio)** |
| Carelon Research | 234.35 | 416.25 | 551.18 | 1.78 | 2.35 | 207.03 | 355.82 | 470.51 | 1.72 | 2.27 |
| CVS Health | 190.55 | 363.01 | 434.57 | 1.91 | 2.28 | 166.72 | 306.90 | 369.11 | 1.84 | 2.21 |
| Optum | 192.96 | 371.54 | 448.26 | 1.93 | 2.32 | 168.13 | 314.92 | 378.07 | 1.87 | 2.25 |

[a] For all three data sources, this includes background rates data through 12/2019

[b] For all three data sources, this includes background rates data through 12/2022

© 2024 Hu M et al. *JAMA Network Open.*

**eTable 8. Sequential Testing Results for the Pediatric Population Receiving BNT162b2 or mRNA-1273 Ancestral Monovalent COVID-19 Vaccines Stratified by Vaccine Dose and Outcome**

| Dose | Age group | BNT162b2 | | | | mRNA-1273 | | | |
|---|---|---|---|---|---|---|---|---|---|
| | | Doses, No. | Outcomes, No. | Person-time, d | Signal status | Doses, No. | Outcomes, No. | Person-time, d | Signal status |
| **Anaphylaxis** | | | | | | | | | |
| Doses 1 and 2 | 6 mo-4 or 5 y | 454 003 | NR | 903 139 | NT | 304 501 | NR | 606 089 | NT |
| | 5 or 6-11 y | 2 715 842 | NR | 5 428 277 | NS | 1687 | 0 | 3346 | NT |
| | 12-15 y | 2 444 395 | NR | 4 887 562 | NS | 1558 | 0 | 3093 | NT |
| | 16-17 y | 1 245 735 | NR | 2 490 728 | NT | 1315 | 0 | 2604 | NT |
| Dose 1 | 6 mo-4 or 5 y | 248 106 | NR | 493 859 | NT | 166 491 | 0 | 331 645 | NT |
| | 5 or 6-11 y | 1 467 128 | NR | 2 932 681 | NT | 1068 | 0 | 2120 | NT |
| | 12-15 y | 1 309 249 | NR | 2 617 844 | NS | 957 | 0 | 1896 | NT |
| | 16-17 y | 676 147 | NR | 1 351 857 | NT | 834 | 0 | 1646 | NT |
| Dose 2 | 6 mo-4 or 5 y | 205 897 | NR | 409 280 | NT | 138 010 | NR | 274 444 | NT |
| | 5 or 6-11 y | 1 248 714 | NR | 2 495 596 | NT | 619 | 0 | 1226 | NT |
| | 12-15 y | 1 135 146 | NR | 2 269 718 | NT | 601 | 0 | 1197 | NT |
| | 16-17 y | 569 588 | 0 | 1 138 871 | NT | 481 | 0 | 958 | NT |
| Dose 3 | 6 mo-4 or 5 y | 102 335 | 0 | 204 292 | NT | 3391 | 0 | 6671 | NT |
| | 5 or 6-11 y | 269 600 | 0 | 538 724 | NT | 725 | 0 | 1448 | NT |
| | 12-15 y | 383 763 | NR | 767 272 | NT | 589 | 0 | 1178 | NT |
| | 16-17 y | 237 024 | NR | 473 794 | NT | 320 | 0 | 640 | NT |
| **Appendicitis** | | | | | | | | | |
| Doses 1 and 2 | 6 mo-4 or 5 y | 351 986 | NR | 11 874 618 | NT | 241 915 | NR | 8 789 224 | NT |
| | 5 or 6-11 y | 2 213 650 | 206 | 72 789 318 | NS | 1212 | 0 | 43 060 | NT |
| | 12-15 y | 2 035 023 | 302 | 66 600 250 | NS | 1187 | 0 | 42 059 | NT |
| | 16-17 y | 1 043 311 | 166 | 34 406 004 | NS | 1013 | 0 | 34 656 | NT |
| Dose 1 | 6 mo-4 or 5 y | 190 648 | NR | 5 463 829 | NT | 131 372 | NR | 4 385 416 | NT |
| | 5 or 6-11 y | 1 182 865 | 92 | 30 910 940 | NS | 755 | 0 | 26 302 | NT |
| | 12-15 y | 1 078 396 | 130 | 27 055 551 | NS | 703 | 0 | 23 821 | NT |
| | 16-17 y | 559 908 | 74 | 14 413 948 | NS | 632 | 0 | 20 186 | NT |
| Dose 2 | 6 mo-4 or 5 y | 161 338 | NR | 6 410 789 | NT | 110 543 | NR | 4 403 808 | NT |
| | 5 or 6-11 y | 1 030 785 | 114 | 41 878 378 | NS | 457 | 0 | 16 758 | NT |
| | 12-15 y | 956 627 | 172 | 39 544 699 | NS | 484 | 0 | 18 238 | NT |
| | 16-17 y | 483 403 | 92 | 19 992 056 | NS | 381 | 0 | 14 470 | NT |
| Dose 3 | 6 mo-4 or 5 y | 83 010 | NR | 3 397 223 | NT | 2845 | 0 | 92 282 | NT |
| | 5 or 6-11 y | 239 487 | 27 | 9 790 542 | NS | 645 | 0 | 23 859 | NT |
| | 12-15 y | 352 339 | 54 | 14 545 967 | NS | 532 | 0 | 20 146 | NT |

© 2024 Hu M et al. *JAMA Network Open.*

| | | | | | | | | | |
|---|---|---|---|---|---|---|---|---|---|
| | 16-17 y | 214 951 | 41 | 8 754 099 | NS | 285 | 0 | 10 536 | NT |
| **Bell palsy** | | | | | | | | | |
| Doses 1and 2 | 6 mo-4 to 5 y | 410 400 | NR | 14 065 149 | NS | 277 737 | NR | 10 222 194 | NS |
| | 5 or 6-11 y | 2 485 011 | 35 | 81 704 667 | NS | 1467 | 0 | 53 845 | NT |
| | 12-15 y | 2 223 492 | 56 | 72 869 846 | NS | 1410 | 0 | 51 392 | NT |
| | 16-17 y | 1 129 439 | 24 | 37 292 252 | NS | 1216 | 0 | 43 061 | NT |
| Dose 1 | 6 mo-4 or 5 y | 221 585 | NR | 6 444 275 | NT | 150 082 | NR | 5 063 381 | NT |
| | 5 or 6-11 y | 1 334 662 | 14 | 35 013 473 | NS | 916 | 0 | 32 855 | NT |
| | 12-15 y | 1 179 765 | 18 | 29 755 101 | NS | 849 | 0 | 29 484 | NT |
| | 16-17 y | 607 786 | NR | 15 737 104 | NS | 760 | 0 | 25 173 | NT |
| Dose 2 | 6 mo-4 or 5 y | 188 815 | NR | 7 620 874 | NT | 127 655 | NR | 5 158 813 | NT |
| | 5 or 6-11 y | 1 150 349 | 22 | 46 691 194 | NS | 551 | 0 | 20 990 | NT |
| | 12-15 y | 1 043 727 | 38 | 43 114 745 | NS | 561 | 0 | 21 908 | NT |
| | 16-17 y | 521 653 | NR | 21 555 148 | NS | 456 | 0 | 17 888 | NT |
| Dose 3 | 6 mo-4 or 5 y | 96 939 | NR | 3 984 353 | NT | 3233 | 0 | 110 964 | NT |
| | 5 or 6-11 y | 259 078 | NR | 10 618 052 | NS | 699 | 0 | 27 077 | NT |
| | 12-15 y | 370 606 | 16 | 15 297 175 | NS | 562 | 0 | 22 080 | NT |
| | 16-17 y | 229 588 | NR | 9 335 942 | NS | 310 | 0 | 12 020 | NT |
| **Common site thrombosis with thrombocytopenia**[a] | | | | | | | | | |
| Doses 1 and 2 | 6 mo-4 or 5 y[b] | 244 267 | 0 | 6 241 793 | NT | 81 661 | 0 | 2 219 079 | NT |
| | 5 or 6-11 y[b] | 2 215 973 | NR | 55 992 946 | NT | 621 | 0 | 16 404 | NT |
| | 12-15 y | 2 038 360 | NR | 51 169 805 | NT | 1188 | 0 | 31 617 | NT |
| | 16-17 y | 1 045 098 | NR | 26 329 445 | NT | 1016 | 0 | 26 702 | NT |
| Dose 1 | 6 mo-4 or 5 y[b] | 131 743 | 0 | 3 201 944 | NT | 43 906 | 0 | 1 196 821 | NT |
| | 5 or 6-11 y[b] | 1 184 106 | NR | 27 642 091 | NT | 398 | 0 | 10 492 | NT |
| | 12-15 y | 1 080 146 | 0 | 24 623 090 | NT | 704 | 0 | 18 802 | NT |
| | 16-17 y | 560 874 | NR | 12 913 395 | NT | 634 | 0 | 16 526 | NT |
| Dose 2 | 6 mo-4 or 5 y[b] | 112 524 | 0 | 3 039 849 | NT | 37 755 | 0 | 1 022 258 | NT |
| | 5 or 6-11 y[b] | 1 031 867 | 0 | 28 350 855 | NT | 223 | 0 | 5912 | NT |
| | 12-15 y | 958 214 | NR | 26 546 715 | NT | 484 | 0 | 12 815 | NT |
| | 16-17 y | 484 224 | NR | 13 416 050 | NT | 382 | 0 | 10 176 | NT |
| Dose 3 | 6 mo-4 or 5 y[b] | 59 404 | 0 | 1 638 795 | NT | 778 | 0 | 19 250 | NT |
| | 5 or 6-11 y[b] | 239 716 | NR | 6 602 510 | NT | 352 | 0 | 9147 | NT |
| | 12-15 y | 352 878 | 0 | 9 765 325 | NT | 533 | 0 | 14 234 | NT |
| | 16-17 y | 215 309 | 0 | 5 886 780 | NT | 285 | 0 | 7596 | NT |
| **Deep vein thrombosis** | | | | | | | | | |
| Doses 1 and 2 | 6 mo-4 or 5 y | 351 932 | NR | 9 020 709 | NT | 241 877 | 0 | 6 579 967 | NT |
| | 5 or 6-11 y | 2 215 893 | NR | 55 988 930 | NT | 1212 | 0 | 32 099 | NT |
| | 12-15 y | 2 038 235 | NR | 51 163 432 | NS | 1188 | 0 | 31 617 | NT |
| | 16-17 y | 1 044 870 | NR | 26 321 797 | NS | 1016 | 0 | 26 702 | NT |
| Dose 1 | 6 mo-4 or 5 y | 190 617 | 0 | 4 651 184 | NT | 131 352 | 0 | 3 581 264 | NT |

© 2024 Hu M et al. *JAMA Network Open.*

| | | | | | | | | | |
|---|---|---|---|---|---|---|---|---|---|
| | 5 or 6-11 y | 1 184 060 | NR | 27 640 017 | NT | 755 | 0 | 20 038 | NT |
| | 12-15 y | 1 080 081 | NR | 24 620 103 | NS | 704 | 0 | 18 802 | NT |
| | 16-17 y | 560 752 | NR | 12 909 608 | NS | 634 | 0 | 16 526 | NT |
| Dose 2 | 6 mo-4 or 5 y | 161 315 | NR | 4 369 525 | NT | 110 525 | 0 | 2 998 703 | NT |
| | 5 or 6-11 y | 1 031 833 | 0 | 28 348 913 | NT | 457 | 0 | 12 061 | NT |
| | 12-15 y | 958 154 | NR | 26 543 329 | NT | 484 | 0 | 12 815 | NT |
| | 16-17 y | 484 118 | NR | 13 412 189 | NT | 382 | 0 | 10 176 | NT |
| Dose 3 | 6 mo-4 or 5 y | 83 015 | 0 | 2 291 244 | NT | 2845 | 0 | 68 640 | NT |
| | 5 or 6-11 y | 239 708 | 0 | 6 601 838 | NT | 648 | 0 | 17 106 | NT |
| | 12-15 y | 352 852 | NR | 9 763 925 | NT | 533 | 0 | 14 234 | NT |
| | 16-17 y | 215 265 | NR | 5 885 237 | NT | 285 | 0 | 7596 | NT |
| **Disseminated intravascular coagulation** | | | | | | | | | |
| Doses 1 and 2 | 6 mo-4 or 5 y | 352 017 | NR | 8 897 561 | NT | 241 928 | 0 | 6 501 186 | NT |
| | 5 or 6-11 y | 2 215 973 | NR | 55 933 013 | NT | 1212 | 0 | 30 713 | NT |
| | 12-15 y | 2 038 363 | 0 | 51 163 245 | NT | 1188 | 0 | 30 749 | NT |
| | 16-17 y | 1 045 113 | 0 | 26 325 916 | NT | 1018 | 0 | 25 832 | NT |
| Dose 1 | 6 mo-4 or 5 y | 190 665 | NR | 4 597 312 | NT | 131 380 | 0 | 3 546 479 | NT |
| | 5 or 6-11 y | 1 184 106 | NR | 27 614 907 | NT | 755 | 0 | 19 448 | NT |
| | 12-15 y | 1 080 148 | 0 | 24 619 938 | NT | 704 | 0 | 18 402 | NT |
| | 16-17 y | 560 883 | 0 | 12 911 429 | NT | 635 | 0 | 15 982 | NT |
| Dose 2 | 6 mo-4 or 5 y | 161 352 | 0 | 4 300 249 | NT | 110 548 | 0 | 2 954 707 | NT |
| | 5 or 6-11 y | 1 031 867 | NR | 28 318 106 | NT | 457 | 0 | 11 265 | NT |
| | 12-15 y | 958 215 | 0 | 26 543 307 | NT | 484 | 0 | 12 347 | NT |
| | 16-17 y | 484 230 | 0 | 13 414 487 | NT | 383 | 0 | 9850 | NT |
| Dose 3 | 6 mo-4 or 5 y | 83 022 | 0 | 2 278 221 | NT | 2845 | 0 | 64 854 | NT |
| | 5 or 6-11 y | 239 717 | 0 | 6 579 046 | NT | 648 | 0 | 16 295 | NT |
| | 12-15 y | 352 881 | NR | 9 759 909 | NT | 533 | 0 | 13 650 | NT |
| | 16-17 y | 215 316 | 0 | 5 884 863 | NT | 285 | 0 | 7132 | NT |
| **Encephalitis or encephalomyelitis** | | | | | | | | | |
| Doses 1 and 2 | 6 mo-4 or 5 y | 410 448 | 0 | 13 758 825 | NT | 277 773 | NR | 10 024 797 | NT |
| | 5 or 6-11 y | 2 485 324 | NR | 81 547 753 | NT | 1469 | 0 | 49 843 | NT |
| | 12-15 y | 2 223 796 | NR | 72 859 944 | NS | 1410 | 0 | 48 910 | NT |
| | 16-17 y | 1 129 627 | NR | 37 287 987 | NT | 1216 | 0 | 40 559 | NT |
| Dose 1 | 6 mo-4 or 5 y | 221 610 | 0 | 6 325 107 | NT | 150 101 | NR | 4 984 704 | NT |
| | 5 or 6-11 y | 1 334 839 | NR | 34 943 578 | NT | 916 | 0 | 30 912 | NT |
| | 12-15 y | 1 179 935 | NR | 29 750 039 | NT | 849 | 0 | 28 438 | NT |
| | 16-17 y | 607 886 | NR | 15 734 035 | NT | 760 | 0 | 23 715 | NT |
| Dose 2 | 6 mo-4 or 5 y | 188 838 | 0 | 7 433 718 | NT | 127 672 | NR | 5 040 093 | NT |
| | 5 or 6-11 y | 1 150 485 | NR | 46 604 175 | NT | 553 | 0 | 18 931 | NT |
| | 12-15 y | 1 043 861 | NR | 43 109 905 | NS | 561 | 0 | 20 472 | NT |
| | 16-17 y | 521 741 | NR | 21 553 952 | NT | 456 | 0 | 16 844 | NT |

© 2024 Hu M et al. *JAMA Network Open.*

| | | | | | | | | | |
|---|---|---|---|---|---|---|---|---|---|
| Dose 3 | 6 mo-4 or 5 y | 96 950 | 0 | 3 945 744 | NT | 3233 | 0 | 101 347 | NT |
| | 5 or 6-11 y | 259 110 | NR | 10 548 412 | NT | 699 | 0 | 24 341 | NT |
| | 12-15 y | 370 652 | 0 | 15 282 789 | NT | 563 | 0 | 20 192 | NT |
| | 16-17 y | 229 634 | 0 | 9 331 463 | NT | 310 | 0 | 10 873 | NT |
| **Guillain-Barré syndrome** | | | | | | | | | |
| Doses 1 and 2 | 6 mo-4 or 5 y | 352 025 | 0 | 11 830 401 | NT | 241 936 | 0 | 8 761 303 | NT |
| Dose 1 | 6 mo-4 or 5 y | 190 670 | 0 | 5 446 456 | NT | 131 384 | 0 | 4 374 485 | NT |
| Dose 2 | 6 mo-4 or 5 y | 161 355 | 0 | 6 383 945 | NT | 110 552 | 0 | 4 386 818 | NT |
| Dose 3 | 6 mo-4 or 5 y | 83 022 | 0 | 3 391 294 | NT | 2845 | 0 | 90 746 | NT |
| **Hemorrhagic stroke** | | | | | | | | | |
| Doses 1 and 2 | 6 mo-4 or 5 y | 351 997 | NR | 8 970 006 | NT | 241 927 | 0 | 6 548 151 | NT |
| Dose 1 | 6 mo-4 or 5 y | 190 654 | NR | 4 629 520 | NT | 131 379 | 0 | 3 567 371 | NT |
| Dose 2 | 6 mo-4 or 5 y | 161 343 | 0 | 4 340 486 | NT | 110 548 | 0 | 2 980 780 | NT |
| Dose 3 | 6 mo-4 or 5 y | 83 017 | 0 | 2 285 757 | NT | 2845 | 0 | 66 970 | NT |
| **Immune thrombocytopenia** | | | | | | | | | |
| Doses 1 and 2 | 6 mo-4 or 5 y | 351 972 | NR | 12 028 216 | NT | 241 891 | NR | 8 887 610 | NS |
| | 5 or 6-11 y | 2 215 661 | 23 | 72 925 239 | NS | 1212 | 0 | 44 796 | NT |
| | 12-15 y | 2 038 029 | 25 | 66 706 657 | NS | 1188 | 0 | 43 176 | NT |
| | 16-17 y | 1 044 876 | 15 | 34 462 183 | NS | 1018 | 0 | 35 938 | NT |
| Dose 1 | 6 mo-4 or 5 y | 190 643 | 0 | 5 522 961 | NT | 131 361 | NR | 4 424 088 | NT |
| | 5 or 6-11 y | 1 183 941 | NR | 30 969 110 | NS | 755 | 0 | 27 077 | NT |
| | 12-15 y | 1 079 968 | NR | 27 098 483 | NS | 704 | 0 | 24 320 | NT |
| | 16-17 y | 560 757 | NR | 14 438 389 | NS | 635 | 0 | 20 921 | NT |
| Dose 2 | 6 mo-4 or 5 y | 161 329 | NR | 6 505 255 | NT | 110 530 | NR | 4 463 522 | NT |
| | 5 or 6-11 y | 1 031 720 | NR | 41 956 129 | NS | 457 | 0 | 17 719 | NT |
| | 12-15 y | 958 061 | NR | 39 608 174 | NS | 484 | 0 | 18 856 | NT |
| | 16-17 y | 484 119 | NR | 20 023 794 | NS | 383 | 0 | 15 017 | NT |
| Dose 3 | 6 mo-4 or 5 y | 83 012 | 0 | 3 415 558 | NT | 2845 | 0 | 97 403 | NT |
| | 5 or 6-11 y | 239 675 | 0 | 9 830 404 | NT | 647 | 0 | 24 998 | NT |
| | 12-15 y | 352 826 | 0 | 14 573 683 | NT | 533 | 0 | 21 004 | NT |
| | 16-17 y | 215 255 | NR | 8 769 474 | NT | 285 | 0 | 11 128 | NT |
| **Myocarditis or pericarditis (all settings) (1-7 d)** | | | | | | | | | |
| Doses 1 and 2 | 6 mo-4 or 5 y | 351 988 | 0 | 2 443 114 | NT | 241 924 | 0 | 1 679 814 | NT |
| | 5 or 6-11 y | 2 215 815 | NR | 15 455 303 | NS | 1212 | 0 | 8392 | NT |
| | 12-15 y | 2 038 160 | 72 | 14 241 393 | S(3) | 1188 | 0 | 8197 | NT |
| | 16-17 y | 1 044 928 | 56 | 7 299 458 | S(3) | 1018 | 0 | 7014 | NT |
| Dose 1 | 6 mo-4 or 5 y | 190 650 | 0 | 1 323 939 | NT | 131 378 | 0 | 912 801 | NT |
| | 5 or 6-11 y | 1 184 019 | NR | 8 261 973 | NT | 755 | 0 | 5244 | NT |
| | 12-15 y | 1 080 046 | NR | 7 547 573 | NS | 704 | 0 | 4859 | NT |

© 2024 Hu M et al. *JAMA Network Open.*

| | | | | | | | | | |
|---|---|---|---|---|---|---|---|---|---|
| | 16-17 y | 560 782 | NR | 3 917 140 | NS | 635 | 0 | 4355 | NT |
| Dose 2 | 6 mo-4 or 5 y | 161 338 | 0 | 1 119 175 | NT | 110 546 | 0 | 767 013 | NT |
| | 5 or 6-11 y | 1 031 796 | NR | 7 193 330 | NS | 457 | 0 | 3148 | NT |
| | 12-15 y | 958 114 | NR | 6 693 820 | S(3) | 484 | 0 | 3338 | NT |
| | 16-17 y | 484 146 | NR | 3 382 318 | S(3) | 383 | 0 | 2659 | NT |
| Dose 3 | 6 mo-4 or 5 y | 83 014 | 0 | 579 161 | NT | 2845 | 0 | 19 413 | NT |
| | 5 or 6-11 y | 239 708 | 0 | 1 672 699 | NT | 648 | 0 | 4517 | NT |
| | 12-15 y | 352 832 | NR | 2 465 354 | S(1) | 533 | 0 | 3719 | NT |
| | 16-17 y | 215 272 | 13 | 1 497 801 | S(2) | 285 | 0 | 1990 | NT |
| **Myocarditis or pericarditis (all settings) (1-21 d)** | | | | | | | | | |
| Doses 1 and 2 | 6 mo-4 or 5 y | 351 988 | NR | 7 252 205 | NT | 241 924 | 0 | 4 987 361 | NT |
| | 5 or 6-11 y | 2 215 815 | 12 | 45 978 229 | NS | 1212 | 0 | 24 750 | NT |
| | 12-15 y | 2 038 160 | 103 | 42 443 102 | S(3) | 1188 | 0 | 24 182 | NT |
| | 16-17 y | 1 044 928 | 71 | 21 746 581 | S(3) | 1018 | 0 | 20 541 | NT |
| Dose 1 | 6 mo-4 or 5 y | 190 650 | 0 | 3 932 631 | NT | 131 378 | 0 | 2 711 909 | NT |
| | 5 or 6-11 y | 1 184 019 | NR | 24 573 073 | NT | 755 | 0 | 15 431 | NT |
| | 12-15 y | 1 080 046 | 19 | 22 468 619 | S(1) | 704 | 0 | 14 352 | NT |
| | 16-17 y | 560 782 | 16 | 11 655 098 | S(1) | 635 | 0 | 12 742 | NT |
| Dose 2 | 6 mo-4 or 5 y | 161 338 | NR | 3 319 574 | NT | 110 546 | 0 | 2 275 452 | NT |
| | 5 or 6-11 y | 1 031 796 | NR | 21 405 156 | NS | 457 | 0 | 9319 | NT |
| | 12-15 y | 958 114 | 84 | 19 974 483 | S(3) | 484 | 0 | 9830 | NT |
| | 16-17 y | 484 146 | 56 | 10 091 483 | S(3) | 383 | 0 | 7799 | NT |
| Dose 3 | 6 mo-4 or 5 y | 83 014 | 0 | 1 728 090 | NT | 2845 | 0 | 54 542 | NT |
| | 5 or 6-11 y | 239 708 | 0 | 4 980 481 | NT | 648 | 0 | 13 186 | NT |
| | 12-15 y | 352 832 | NR | 7 349 009 | S(1) | 533 | 0 | 10 924 | NT |
| | 16-17 y | 215 272 | 14 | 4 436 604 | S(1) | 285 | 0 | 5871 | NT |
| **Myocarditis or pericarditis (IP, OP, and ED) (1-7 d)** | | | | | | | | | |
| Doses 1 and 2 | 6 mo-4 or 5 y[b] | 352 019 | 0 | 2 427 241 | NT | 152 637 | 0 | 1 053 032 | NT |
| | 5 to 6-11 y[b] | 2 215 967 | NR | 15 450 396 | NT | 621 | 0 | 4208 | NT |
| | 12-15 y | 2 038 335 | 55 | 14 242 747 | S(3) | 1188 | 0 | 8114 | NT |
| | 16-17 y | 1 045 077 | 40 | 7 300 604 | S(3) | 1018 | 0 | 6924 | NT |
| Dose 1 | 6 mo-4 or 5 y[b] | 190 667 | 0 | 1 316 715 | NT | 82 934 | 0 | 573 125 | NT |
| | 5 or 6-11 y[b] | 1 184 102 | NR | 8 259 674 | NT | 398 | 0 | 2691 | NT |
| | 12-15 y | 1 080 136 | NR | 7 548 270 | NT | 704 | 0 | 4819 | NT |
| | 16-17 y | 560 863 | NR | 3 917 764 | NS | 635 | 0 | 4297 | NT |
| Dose 2 | 6 mo-4 or 5 y[b] | 161 352 | 0 | 1 110 526 | NT | 69 703 | 0 | 479 907 | NT |
| | 5 or 6-11 y[b] | 1 031 865 | NR | 7 190 722 | NT | 223 | 0 | 1517 | NT |
| | 12-15 y | 958 199 | NR | 6 694 477 | S(3) | 484 | 0 | 3295 | NT |
| | 16-17 y | 484 214 | NR | 3 382 840 | S(3) | 383 | 0 | 2627 | NT |
| Dose 3 | 6 mo-4 or 5 y[b] | 83 021 | 0 | 578 025 | NT | 1185 | 0 | 7882 | NT |
| | 5 or 6-11 y[b] | 239 718 | 0 | 1 671 051 | NT | 352 | 0 | 2405 | NT |

© 2024 Hu M et al. *JAMA Network Open.*

| | | | | | | | | | |
|---|---|---|---|---|---|---|---|---|---|
| | 12-15 y | 352 870 | NR | 2 465 157 | S(1) | 533 | 0 | 3707 | NT |
| | 16-17 y | 215 311 | NR | 1 498 007 | S(2) | 285 | 0 | 1966 | NT |
| **Myocarditis or pericarditis (IP, OP, and ED) (1-21 d)** | | | | | | | | | |
| Doses 1 and 2 | 6 mo-4 or 5 y[b] | 352 019 | 0 | 7 198 601 | NT | 152 637 | 0 | 3 125 323 | NT |
| | 5 or 6-11 y[b] | 2 215 967 | NR | 45 959 404 | NT | 621 | 0 | 12 298 | NT |
| | 12-15 y | 2 038 335 | 61 | 42 446 709 | S(3) | 1188 | 0 | 23 893 | NT |
| | 16-17 y | 1 045 077 | 46 | 21 749 769 | S(3) | 1018 | 0 | 20 217 | NT |
| Dose 1 | 6 mo-4 or 5 y[b] | 190 667 | 0 | 3 908 994 | NT | 82 934 | 0 | 1 702 556 | NT |
| | 5 or 6-11 y[b] | 1 184 102 | NR | 24 564 532 | NT | 398 | 0 | 7883 | NT |
| | 12-15 y | 1 080 136 | NR | 22 470 533 | NS | 704 | 0 | 14 199 | NT |
| | 16-17 y | 560 863 | 12 | 11 656 804 | S(1) | 635 | 0 | 12 513 | NT |
| Dose 2 | 6 mo-4 or 5 y[b] | 161 352 | 0 | 3 289 607 | NT | 69 703 | 0 | 1 422 767 | NT |
| | 5 or 6-11 y[b] | 1 031 865 | NR | 21 394 872 | NT | 223 | 0 | 4415 | NT |
| | 12-15 y | 958 199 | NR | 19 976 176 | S(3) | 484 | 0 | 9694 | NT |
| | 16-17 y | 484 214 | 35 | 10 092 965 | S(3) | 383 | 0 | 7704 | NT |
| Dose 3 | 6 mo-4 or 5 y[b] | 83 021 | 0 | 1 723 572 | NT | 1185 | 0 | 22 367 | NT |
| | 5 or 6-11 y[b] | 239 718 | 0 | 4 973 111 | NT | 352 | 0 | 6892 | NT |
| | 12-15 y | 352 870 | NR | 7 348 086 | S(1) | 533 | 0 | 10 753 | NT |
| | 16-17 y | 215 311 | NR | 4 437 033 | S(1) | 285 | 0 | 5721 | NT |
| **Narcolepsy** | | | | | | | | | |
| Doses 1 and 2 | 5 or 6-11 y | 2 215 928 | NR | 72 934 272 | NS | 1212 | 0 | 44 796 | NT |
| | 12-15 y | 2 038 107 | 16 | 66 709 380 | NS | 1188 | 0 | 43 176 | NT |
| | 16-17 y | 1 044 699 | 24 | 34 456 767 | NS | 1017 | 0 | 35 896 | NT |
| Dose 1 | 5 or 6-11 y | 1 184 080 | NR | 30 972 795 | NT | 755 | 0 | 27 077 | NT |
| | 12-15 y | 1 080 018 | NR | 27 099 946 | NS | 704 | 0 | 24 320 | NT |
| | 16-17 y | 560 647 | NR | 14 435 556 | NT | 635 | 0 | 20 921 | NT |
| Dose 2 | 5 or 6-11 y | 1 031 848 | NR | 41 961 477 | NS | 457 | 0 | 17 719 | NT |
| | 12-15 y | 958 089 | NR | 39 609 434 | NS | 484 | 0 | 18 856 | NT |
| | 16-17 y | 484 052 | NR | 20 021 211 | NS | 382 | 0 | 14 975 | NT |
| Dose 3 | 5 or 6-11 y | 239 715 | 0 | 9 832 012 | NT | 648 | 0 | 25 021 | NT |
| | 12-15 y | 352 841 | NR | 14 574 277 | NS | 533 | 0 | 21 004 | NT |
| | 16-17 y | 215 221 | 12 | 8 768 192 | NS | 285 | 0 | 11 128 | NT |
| **Nonhemorrhagic stroke** | | | | | | | | | |
| Doses 1 and 2 | 6 mo-4 or 5 y | 352 009 | 0 | 8 942 566 | NT | 241 930 | NR | 6 530 082 | NT |
| | 5 or 6-11 y | 2 215 960 | NR | 55 955 653 | NT | 1212 | 0 | 31 259 | NT |
| | 12-15 y | 2 038 354 | 0 | 51 165 669 | NT | 1188 | 0 | 31 151 | NT |
| | 16-17 y | 1 045 115 | NR | 26 327 584 | NT | 1018 | 0 | 26 192 | NT |
| Dose 1 | 6 mo-4 or 5 y | 190 661 | 0 | 4 617 655 | NT | 131 381 | NR | 3 559 300 | NT |
| | 5 or 6-11 y | 1 184 098 | NR | 27 625 211 | NT | 755 | 0 | 19 695 | NT |
| | 12-15 y | 1 080 143 | 0 | 24 621 154 | NT | 704 | 0 | 18 574 | NT |
| | 16-17 y | 560 884 | NR | 12 912 371 | NT | 635 | 0 | 16 176 | NT |

© 2024 Hu M et al. *JAMA Network Open.*

| | | | | | | | | | |
|---|---|---|---|---|---|---|---|---|---|
| Dose 2 | 6 mo-4 or 5 y | 161 348 | 0 | 4 324 911 | NT | 110 549 | 0 | 2 970 782 | NT |
| | 5 or 6-11 y | 1 031 862 | 0 | 28 330 442 | NT | 457 | 0 | 11 564 | NT |
| | 12-15 y | 958 211 | 0 | 26 544 515 | NT | 484 | 0 | 12 577 | NT |
| | 16-17 y | 484 231 | NR | 13 415 213 | NT | 383 | 0 | 10 016 | NT |
| Dose 3 | 6 mo-4 or 5 y | 83 019 | NR | 2 282 885 | NT | 2845 | 0 | 66 156 | NT |
| | 5 or 6-11 y | 239 722 | 0 | 6 587 840 | NT | 648 | 0 | 16 549 | NT |
| | 12-15 y | 352 881 | 0 | 9 761 993 | NT | 533 | 0 | 13 822 | NT |
| | 16-17 y | 215 317 | NR | 5 885 718 | NT | 285 | 0 | 7299 | NT |
| **Pulmonary embolism** | | | | | | | | | |
| Doses 1 and 2 | 6 mo-4 or 5 y[b] | 239 532 | 0 | 6 178 317 | NT | 160 273 | 0 | 4 377 689 | NT |
| | 5 or 6-11 y | 2 215 976 | 0 | 56 012 340 | NT | 1212 | 0 | 32 579 | NT |
| | 12-15 y | 2 038 334 | NR | 51 168 510 | NT | 1188 | 0 | 31 876 | NT |
| | 16-17 y | 1 044 978 | NR | 26 326 099 | NS | 1018 | 0 | 27 082 | NT |
| Dose 1 | 6 mo-4 or 5 y[b] | 130 125 | 0 | 3 196 420 | NT | 87 477 | 0 | 2 391 693 | NT |
| | 5 or 6-11 y | 1 184 108 | 0 | 27 650 979 | NT | 755 | 0 | 20 295 | NT |
| | 12-15 y | 1 080 133 | 0 | 24 622 518 | NT | 704 | 0 | 18 935 | NT |
| | 16-17 y | 560 809 | NR | 12 911 833 | NS | 635 | 0 | 16 775 | NT |
| Dose 2 | 6 mo-4 or 5 y[b] | 109 407 | 0 | 2 981 897 | NT | 72 796 | 0 | 1 985 996 | NT |
| | 5 or 6-11 y | 1 031 868 | 0 | 28 361 361 | NT | 457 | 0 | 12 284 | NT |
| | 12-15 y | 958 201 | NR | 26 545 992 | NT | 484 | 0 | 12 941 | NT |
| | 16-17 y | 484 169 | NR | 13 414 266 | NT | 383 | 0 | 10 307 | NT |
| Dose 3 | 6 mo-4 or 5 y[b] | 55 239 | 0 | 1 527 770 | NT | 2067 | 0 | 50 277 | NT |
| | 5 or 6-11 y | 239 722 | NR | 6 610 019 | NT | 648 | 0 | 17 308 | NT |
| | 12-15 y | 352 879 | 0 | 9 766 897 | NT | 533 | 0 | 14 393 | NT |
| | 16-17 y | 215 293 | NR | 5 886 669 | NT | 285 | 0 | 7721 | NT |
| **Seizure[c]** | | | | | | | | | |
| Doses 1 and 2 | 6 mo-1 y | 450 140 | 25 | 1 191 471 | NS | 302 275 | 15 | 872 958 | NS |
| | 2-4 or 5 y | 450 140 | 36 | 2 373 696 | S(3) | 302 275 | 29 | 1 522 577 | S(2) |
| | 5 to 6-11 y | 2 692 535 | 71 | 21 462 208 | NS | 1667 | 0 | 13 078 | NT |
| | 12-15 y | 2 430 285 | 52 | 19 407 961 | NS | 1545 | 0 | 12 180 | NT |
| | 16-17 y | 1 238 940 | 38 | 9 890 880 | NS | 1300 | 0 | 10 220 | NT |
| Dose 1 | 6 mo-1 y | 245 400 | 14 | 660 359 | NS | 164 942 | NR | 486 994 | NS |
| | 2-4 or 5 y | 245 400 | 21 | 1 284 771 | S(2) | 164 942 | NR | 821 447 | NS |
| | 5 or 6-11 y | 1 454 544 | 41 | 11 598 717 | NS | 1051 | 0 | 8240 | NT |
| | 12-15 y | 1 301 389 | 27 | 10 393 770 | NS | 947 | 0 | 7458 | NT |
| | 16-17 y | 671 951 | 24 | 5 363 845 | NS | 821 | 0 | 6431 | NT |
| Dose 2 | 6 mo-1 y | 204 740 | 11 | 531 112 | NS | 137 333 | NR | 385 964 | NS |
| | 2-4 or 5 y | 204 740 | 15 | 1 088 925 | S(2) | 137 333 | NR | 701 130 | S(2) |
| | 5 or 6-11 y | 1 237 991 | 30 | 9 863 491 | NS | 616 | 0 | 4838 | NT |
| | 12-15 y | 1 128 896 | 25 | 9 014 191 | NS | 598 | 0 | 4722 | NT |
| | 16-17 y | 566 989 | 16 | 4 527 035 | NS | 479 | 0 | 3789 | NT |

© 2024 Hu M et al. *JAMA Network Open.*

| | | | | | | | | | |
|---|---|---|---|---|---|---|---|---|---|
| Dose 3 | 6 mo-1 y | 102 099 | NR | 231 518 | NT | 3376 | NR | 8115 | NT |
| | 2-4 or 5 y | 102 099 | NR | 581 953 | NS | 3376 | 0 | 18 177 | NT |
| | 5 or 6-11 y | 268 990 | NR | 2 145 055 | NS | 725 | 0 | 5768 | NT |
| | 12-15 y | 382 249 | NR | 3 052 406 | NS | 587 | 0 | 4684 | NT |
| | 16-17 y | 236 352 | NR | 1 879 834 | NS | 318 | 0 | 2532 | NT |

Abbreviations: ED, emergency department; IP, inpatient; NR, not reported (data for sample sizes of 1 to 10 and that can be used to back-calculate small cell sizes were masked for confidentiality); NS, test was initiated (cases >3) but no statistical signal was observed; NT, test was not initiated due to low outcome counts (cases <3); OP, outpatient; S, test met the statistical threshold for a signal (the number in paratheses is the count of databases that had a statistical signal for that specific outcome, dose, age group, and brand).

[a]Common site thrombosis with thrombocytopenia is combined with unusual site thrombosis (broad) with thrombocytopenia consisting of a thrombotic event (made up of other events, such as acute myocardial infarction and deep vein thrombosis) and a thrombocytopenia event (defined in the IP, OP, and physician billing setting). The overall setting definition for the outcome depends on individual setting definitions for each of these components.

[b]For these outcome-age combinations, testing occurred in fewer than 3 data sources because 1 or more data source did not have available background rates; for these outcome-age combinations, number of doses are reported for the data sources for which sequential testing data were available.

[c]For the outcome seizure, the number of doses reported in the age groups 6 months to 1 year and 2 to 4 or 5 years corresponds to the total number of doses monitored descriptively in the age groups 6 months to 4 or 5 years; all other statistics from sequential testing (number of outcomes, person-time, and statistical signal status) are reported for the corresponding age stratifications listed in the table above due to availability of background rates for those age strata. Sequential testing ended earlier in the age groups 5 or 6 to 17 years due to limited accrual of new exposures; therefore, the data cuts used are different from the age group 6 months to 4 or 5 years. For the age group 5 or 6 to 17 years, Optum data through April 2023, Carelon Research data through January 2023, and CVS Health data through December 2022 were used. For the age group 6 months to 4 or 5 years, Optum data through January 2023, Carelon Research data through March 2023, and CVS Health data through February 2023 were used.

© 2024 Hu M et al. *JAMA Network Open.*

**eFigure. Distribution of Days Between Vaccination and Seizures (N =72 Seizures Events) After Monovalent BNT162b2 (2-4 years) and mRNA-1273 (Ages 2-5 years) COVID-19 Vaccines[a]**

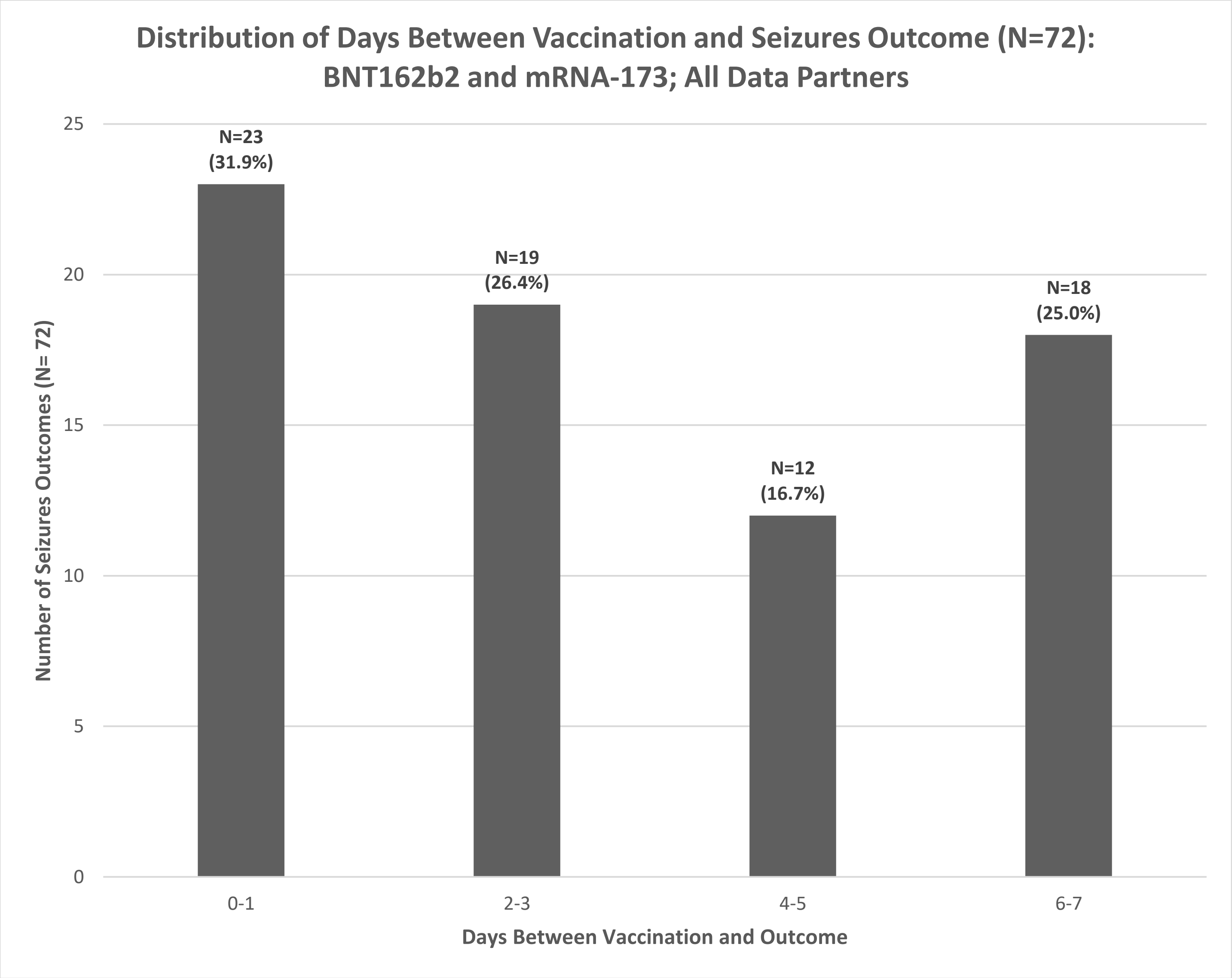


[a] Combines data for all three data sources: Carelon Research: Data through 3/2023, CVS Health: Data through 2/2023, Optum: Data through 4/2023

© 2024 Hu M et al. *JAMA Network Open.*

**eAppendix. Medical Record Review**

Medical record review was conducted for the myocarditis/pericarditis outcome following identification of a statistical signal. Brighton Collaboration case definitions were used to adjudicate cases.[39] Records meeting the confirmed or probable Brighton classifications were considered true myocarditis/pericarditis cases for the validation analyses.

Of the 153 cases of myocarditis/pericarditis after COVID-19 vaccination among children aged 12-17 years, medical record review was conducted for a sample of 40 cases whose records could be obtained. Twenty-nine of these cases (72.5%) were confirmed as true cases of myocarditis/pericarditis, of which 27 patients were male, and 19 were hospitalized with a median length of hospital stay of 2 days (interquartile range: 1, 3). The median time from vaccination to presentation of myocarditis/pericarditis event was 3 days (interquartile range: 2, 5). Medical record review and further validation efforts for seizure are currently underway.

© 2024 Hu M et al. *JAMA Network Open.*

**eReferences**


1. U.S. Food and Drug Administration. (2022, June 16). Pfizer-BioNTech COVID-19 Vaccine EUA Amendment for Use in Individuals 6 Months Through 4 Years of Age. U.S. Food and Drug Administration. Retrieved July 27, 2022, from https://www.fda.gov/media/159393/download

2. U.S. Food and Drug Administration. (2022, January 3). Coronavirus (COVID-19) Update: FDA Takes Multiple Actions to Expand use of Pfizer-BioNtech COVID-19 vaccine. U.S. Food and Drug Administration. Retrieved July 27, 2022, from https://www.fda.gov/news-events/press-announcements/coronavirus-covid-19-update-fda-takes-multiple-actions-expand-use-pfizer-biontech-covid-19-vaccine

3. U.S. Food and Drug Administration. (2022, May 17). Coronavirus (COVID-19) Update: FDA Expands Eligibility for Pfizer-BioNtech COVID-19 Vaccine Booster Dose to Children 5 through 11 Years. U.S. Food and Drug Administration. Retrieved July 27, 2022, from https://www.fda.gov/news-events/press-announcements/coronavirus-covid-19-update-fda-expands-eligibility-pfizer-biontech-covid-19-vaccine-booster-dose

4. U.S. Food and Drug Administration. (2021, May 10). Emergency Use Authorization (EUA) Amendment for an Unapproved Product Review Memorandum. U.S. Food and Drug Administration. Retrieved July 27, 2022, from https://www.fda.gov/media/148542/download

5. Cho, D. (2021, August 12). Review Memorandum. U.S. Food and Drug Administration. Retrieved July 27, 2022, from https://www.fda.gov/media/151613/download

6. O'Shaughnessy, J. A. (2022, July 8). Pfizer-BioNTech COVID-19 Vaccine EUA LOA reissued. U.S. Food and Drug Administration. Retrieved July 27, 2022, from https://www.fda.gov/media/150386/download

7. U.S. Food and Drug Administration. (2021, August 23). FDA Approves First COVID-19 Vaccine. U.S. Food and Drug Administration. Retrieved July 27, 2022, from https://www.fda.gov/news-events/press-announcements/fda-approves-first-covid-19-vaccine

8. O'Shaughnessy, J. A. (2022, June 17). Moderna COVID-19 Vaccine EUA Letter of Authorization. U.S. Food and Drug Administration. Retrieved July 27, 2022, from https://www.fda.gov/media/144636/download

9. U.S. Food and Drug Administration. (2022, August 19). FDA Roundup: August 19, 2022. U.S. Food and Drug Administration. Retrieved December 8, 2022, from https://www.fda.gov/news-events/press-announcements/fda-roundup-august-19-2022

10. Al Qudah Z, Abukwaik W,  Souayah N. Stroke after Vaccination in United States. A Report from the CDC/FDA Vaccine Adverse Event Reporting System. [1990–2010] (P01.009). Neurology. 2012; 78(Meeting Abstracts 1): P01.009-P01.009.

11. Smeeth L, Thomas SL, Hall AJ, Hubbard R, Farrington P, Vallance P. Risk of myocardial infarction and stroke after acute infection or vaccination. New England Journal of Medicine. 2004; 351(25): 2611-2618.

12. Rüggeberg JU, Gold MS, Bayas J-M, et al., Anaphylaxis: case definition and guidelines for data collection, analysis, and presentation of immunization safety data. Vaccine. 2007; 25(31): 5675-5684.

13. Su JR, Moro PL, Ng CS, Lewis PW, Said MA, Cano MV. Anaphylaxis after vaccination reported to the Vaccine Adverse Event Reporting System, 1990-2016. Journal of Allergy and Clinical Immunology. 2019; 143(4): 1465-1473.

14. Donahue JG, Kieke BA, Lewis EM, et al. Near Real-Time Surveillance to Assess the Safety of the 9-Valent Human Papillomavirus Vaccine. Pediatrics. 2019; 144(6): e20191808.


© 2024 Hu M et al. *JAMA Network Open.*

15. GeeJ, Naleway A, Shui I et al. Monitoring the safety of quadrivalent human papillomavirus vaccine: findings from the Vaccine Safety Datalink. Vaccine. 2011; 29(46): 8279-84.

16. Renoud L, Khouri C, Revol B, et al. Association of Facial Paralysis With mRNA COVID-19 Vaccines: A Disproportionality Analysis Using the World Health Organization Pharmacovigilance Database. JAMA Internal Medicine. 2021; 181(9): 1243–1245.

17. Pishko AM, Bussel JB, Cines DB. COVID-19 vaccination and immune thrombocytopenia. Nature Medicine. 2021; 27: 1145–1146 .

18. Kearon C. Natural history of venous thromboembolism. Circulation. 2003; 107(23_suppl_1): I-22-I-30.

19. Vickers E.R, McClure DL, Naleway AL, et al. Risk of venous thromboembolism following influenza vaccination in adults aged 50 years and older in the Vaccine Safety Datalink. Vaccine. 2017; 35(43): 5872-5877.

20. Tang N, Li D, Wang X, Sun Z . Abnormal coagulation parameters are associated with poor prognosis in patients with novel coronavirus pneumonia. Journal of Thrombosis Haemostasis. 2020; 18(4): 844-847.

21. Pellegrino P, Carnovale C, Perrone V, et al. Acute disseminated encephalomyelitis onset: evaluation based on vaccine adverse events reporting systems. PloS one. 2013; 8(10): e77766.

22. Dodd CN, Romio SA, Black S, et al. International collaboration to assess the risk of Guillain Barré Syndrome following Influenza A (H1N1) 2009 monovalent vaccines. Vaccine. 2013; 31(40): 4448-4458.

23. Schonberger LB, Bregman DJ, Sullivan-Bolyai JZ, et al. Guillain-Barré syndrome following vaccination in the national influenza immunization program, United States, 1976–1977. American Journal of Epidemiology. 1979; 110(2): 105-123.

24. Black C, Kaye JA, Jick H. MMR vaccine and idiopathic thrombocytopaenic purpura. British Journal of Clinical Pharmacology. 2003; 55(1): 107-111.

25. D'alò GL, Zorzoli E, Capanna A, et al. Frequently asked questions on seven rare adverse events following immunization. Journal of Preventive Medicine Hygiene. 2017; 58(1): E13.

26. Hennon TR, Penque MD, Abdul-Aziz R, et al. COVID-19 associated Multisystem Inflammatory Syndrome in Children (MIS-C) guidelines; a Western New York approach. Progress in Pediatric Cardiology. 2020; 57: 1-6.

27. Oster ME, Shay DK, Su JR, et al. Myocarditis Cases Reported After mRNA-Based COVID-19 Vaccination in the US From December 2020 to August 2021. JAMA. 2022; 327(4): 331–340.

28. Klein N. Myocarditis Analyses in the Vaccine Safety Datalink: Rapid Cycle Analyses and “Head-to-Head” Product Comparisons. Presentation at: Advisory Committee on Immunization Practices; October, 2021.

29. Duffy J, Weintraub E, Vellozzi C, DeStefano F . Narcolepsy and influenza A (H1N1) pandemic 2009 vaccination in the United States. Neurology. 2014; 83(20): 1823-1830.

30. Montplaisir J, Petit D, Quinn M-J, et al. Risk of narcolepsy associated with inactivated adjuvanted (AS03) A/H1N1 (2009) pandemic influenza vaccine in Quebec. PloS one. 2014; 9(9): e108489.

31. Sarkanen TO, Alakuijala APE, Dauvilliers YA, Partinen MM. Incidence of narcolepsy after H1N1 influenza and vaccinations: Systematic review and meta-analysis. Sleep Medicine Reviews. 2018; 38: 177-186.

32. Kearon C, Akl EA. Duration of anticoagulant therapy for deep vein thrombosis and pulmonary embolism. Blood. 2014; 123(12): 1794-1801.

© 2024 Hu M et al. *JAMA Network Open.*

33. Duffy J, Weintraub E, Hambidge SJ, et al. Febrile seizure risk after vaccination in children 6 to 23 months. Pediatrics. 2016; 138(1). e20160320.

34. Agmon-Levin N, Kivity S, Szyper-Kravitz M, Shoenfeld Y., Transverse myelitis and vaccines: a multi-analysis. Lupus. 2009; 18(13): 1198-1204.

35. Whitworth H, Sartain SE, Kumar R, et al. Rate of thrombosis in children and adolescents hospitalized with COVID-19 or MIS-C. Blood. 2021; 138(2): 190-198.

36. License for use of current procedural terminology, Fourth edition (‘Cpt®’) CMS.gov. Available at: https://www.cms.gov/license/ama?file=%2Ffiles%2Fzip%2Flist-codes-effective-january-1-2023-published-december-1-2022.zip (Accessed: 31 August 2023).

37. IIS (2023) Centers for Disease Control and Prevention. Available at: https://www2a.cdc.gov/vaccines/iis/iisstandards/vaccines.asp?rpt=cvx (Accessed: 31 August 2023).

38. (2023) National Drug Code Directory. Available at: https://www.accessdata.fda.gov/scripts/cder/ndc/index.cfm (Accessed: 06 October 2023).

39. Sexson Tejtel, S. K., Munoz, F. M., Al-Ammouri, I., Savorgnan, F., Guggilla, R. K.,Khuri-Bulos, N., Phillips, L., & Engler, R. (2022). Myocarditis and pericarditis: Case definition and guidelines for data collection, analysis, and presentation of immunization safety data. Vaccine, 40(10), 1499–1511. https://doi.org/10.1016/j.vaccine.2021.11.074


© 2024 Hu M et al. *JAMA Network Open.*
