# Supplementary material for: Safety of Ancestral Monovalent BNT162b2, mRNA-1273, and NVX-CoV2373 COVID-19 Vaccines in US Children Aged 6 Months to 17 Years
Source: JAMA Netw Open. 2024 Apr 24;7(4):e248192. doi: 10.1001/jamanetworkopen.2024.8192 (PMC11043896; doi:10.1001/jamanetworkopen.2024.8192)
Supplement: Supplement 2. — Data Sharing Statement [file jamanetwopen-e248192-s002.pdf]

Data Sharing Statement

Hu. Safety of Ancestral Monovalent BNT162b2, mRNA-1273, and NVX-CoV2373 COVID-19 Vaccines in US Children Aged 6 Months to 17 Years. *JAMA Netw Open*. Published April 24, 2024. doi:10.1001/jamanetworkopen.2024.8192

Data
Data available: No

Additional Information
Explanation for why data not available: FDA receives aggregate data of results from directed analyses and the original patient level claims data are retained by health plans to preserve individual patient privacy. We can share aggregated results information on fields in the data set if requested.
